# Polyphenolic Composition and Anti-Melanoma Activity of White Forsythia (*Abeliophyllum distichum* Nakai) Organ Extracts

**DOI:** 10.3390/plants9060757

**Published:** 2020-06-17

**Authors:** Tong-Kewn Yoo, Ju-Sung Kim, Tae Kyung Hyun

**Affiliations:** 1 Department of Industrial Plant Science and Technology, Chungbuk National University, Cheongju 28644, Korea; ydk31197586@gmail.com; 2 College of Agriculture & Life Sciences, SARI, Jeju National University, Jeju 63243, Korea; aha2011@jejunu.ac.kr

**Keywords:** *Abeliophyllum distichum*, anti-melanoma, apoptosis, differentially expressed gene, polyphenolic compound

## Abstract

*Abeliophyllum distichum* Nakai, commonly called white forsythia, is a monotypic genus endemic to Korea. Although *A. distichum* is mainly used as an ornamental plant because of its horticultural value, recent studies have demonstrated its bioactivities, including antioxidant and anti-inflammatory activities, prompting us to investigate the potential anticancer effect of *A. distichum* organ extracts (leaves, fruit, and branches) against human melanoma SK-MEL-2 cells. The methanol extract of *A. distichum* leaves (AL) exhibited dose- and time-dependent cytotoxicities against SK-MEL-2 cells but not against HDFa human dermal fibroblasts. Based on high-performance liquid chromatography analysis, we identified 18 polyphenolic compounds from *A. distichum* organ extracts and suggest that differences in anticancer activity between organ extracts should be caused by different compositions of polyphenolic compounds. Additionally, the Annexin V/propidium iodide staining assay and analysis of caspase activity and expression indicated that AL induced cell death, including early and late apoptosis, as well as necrosis, by inducing the extrinsic pathway. Furthermore, we analyzed the differentially expressed genes between mock- and AL-treated cells using RNA-seq technology, suggesting that the anti-melanoma action of AL is mediated by down-regulation of the phosphoinositide 3-kinase (PI3K)/Akt signaling pathway. Taken together, these results shed light on the potential use of *A. distichum* as a green resource with potent anti-melanoma activity.

## 1. Introduction

Malignant melanoma is the deadliest skin cancer type that originates from pigment-producing cells known as melanocytes [1]. It is caused by multiple and progressive DNA damage related to proto-oncogene activation, down-regulation of tumor suppressor genes, and structural changes of the chromosomes [2]. The etiology of all cancers depends on the interplay between genetic and environmental factors [3], and approximately 60–70% of malignant melanomas are presumed to be caused by ultraviolet (UV) radiation exposure [4]. Since malignant melanomas have been treated by surgical removal, cryosurgery, radiation therapy, or chemotherapy, there has been a growing interest in the use of complementary and alternative medicines because of the disadvantages mediated by conventional cancer chemotherapies and the health benefits of phytochemicals [5]. Different mechanisms and pathways are regulated by the anti-melanoma activity of phytochemicals, such as increased apoptosis, caspase activity, and expression of *p53* and Bcl-2-associated X protein (*bax*), and the inhibition of angiogenesis and tumor-promoting proteins [5]. The functional diversity of phytochemicals should provide unique and renewable resources to discover and develop potential new antitumor agents, suggesting that the investigation of biological and pharmaceutical properties of medicinal plants and their natural products is an important issue in the development of complementary or adjunctive therapies.

*Abeliophyllum distichum* Nakai is a deciduous flowering shrub, a monotypic genus with a single species of deciduous shrub in the olive family [6]. Although *A. distichum* has been used as a horticultural crop because of its ornamental value, the pharmaceutical properties of *A. distichum* extracts have been recently revealed, such as the anti-inflammatory effect [7], antioxidant activity [6], DNA damage inhibition [8], whitening property [9], anti-diabetic effect by inhibiting aldose reductase [10], antihypertensive activity [11], and anti-proliferative activity against human colorectal cancer cells [12]. Additionally, phytochemical investigations of *A. distichum* have revealed the presence of multiple active ingredients, such as acteoside, eutigoside B, isoacteoside, rutin, cornoside, hirsutrin, chlorogenic acid, caffeic acid, gentisic acid, ferulic acid, and quercetin [6,10,11], indicating the potential of *A. distichum* to be developed as a phytomedicine and a source to develop anti-melanoma agents, although no systematic study exists regarding the anti-melanoma action of *A. distichum*.

Therefore, in this study, the methanol extracts of *A. distichum* fruit, branches, and leaves were analyzed to assess the anti-melanoma effect of *A. distichum*, as well as to identify and quantify polyphenolic compounds, using high-performance liquid chromatography (HPLC). Additionally, to investigate the possible mechanism of anti-melanoma action of *A. distichum* extract, we performed pathway enrichment analysis of the differentially expressed genes (DEGs) in response to *A. distichum* extract.

## 2. Results

### 2.1. Effects of A. distichum Organ Extracts on the Viability of Human Melanoma SK-MEL-2 Cells

**Figure 1:**
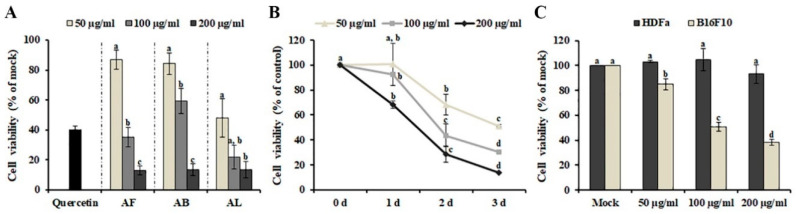
Anti-melanoma effect of methanol extracts obtained from *Abeliophyllum distichum* leaves (AL), fruit (AF), and branches (AB). (**A**) Effect of *A. distichum* organ extracts on the cell viability of SK-MEL-2 cells. SK-MEL-2 cells were treated with different concentrations of each extract for 48 h, and cell viability was determined using MTT [3-(4,5-dimethylthiazol-2-yl)-2,5-diphenyltetrazolium bromide] assay. 10 μM of Quercetin was used as positive control. (**B**) Time and dose dependence of the anti-melanoma effect induced by AL. (**C**) Effect of AL on the cell viability of B16F10 murine melanoma cells and HDFa human dermal fibroblast cells. B16F10 cells and HDFa cells were treated with the indicated doses of AL for 48 h, and the viabilities of the cells were determined. The values are the means of three independent experiments, and the error bars represent the standard error. Values in the same column with different superscript letters are significantly different (*p* < 0.05).

The effect of the methanol extracts of leaves, branches, and fruit on the growth of human SK-MEL-2 melanoma cells was investigated using MTT [3-(4,5-dimethylthiazol-2-yl)-2,5-diphenyltetrazolium bromide] assay. As shown in Figure 1A, incubation with 50 μg/ml of *A. distichum* leaves (AL) (48.091 ± 12.741%) strongly inhibited the proliferation of SK-MEL-2 cells compared with *A. distichum* branches (AB) (84.229 ± 7.335%) and *A. distichum* fruits (AF) (86.949 ± 6.287%), although 200 μg/ml of all extracts showed similar cytotoxic activity. Therefore, AL was selected for further experiments. AL exhibited a dose- and time-dependent inhibitory effect on the viability of SK-MEL-2 cells (Figure 1B). Although chemotherapeutic agents, including irinotecan, doxorubicin, oxaliplatin, and cyclophosphamide, have shown promising results alone or in combination with other cancer therapies, their unexpected activities against normal cells have led to harmful side effects [13]. Therefore, identifying and developing tumor-specific agents remains an outstanding challenge in cancer therapy. To investigate the effect of AL on the viability of normal cells and other melanoma cells, murine melanoma (B16F10) cells and adult human dermal fibroblasts (HDFa) were treated with different concentrations (50 μg/ml, 100 μg/ml and 200 μg/ml) of AL for 48 h. As shown in Figure 1C, AL showed cytotoxic effects on B16F10 cells at concentrations of 50–200 μg/ml, but it was not cytotoxic against HDFa, even at the maximum tested concentrations. These findings indicate that AL can be a potent source of anticancer agents because of its selective cytotoxicity against melanoma cells and lack of toxicity in non-cancerous cells.

### 2.2. Composition of Polyphenolic Compounds in A. distichum Organ Extracts

Polyphenolic compounds, including flavonoids and tannins, are beneficial plant compounds that offer various health benefits and are important anticancer agents [14]. Additionally, they are potential active compounds in *A. distichum* extract. To identify the active polyphenolic compounds that potentially exhibit cytotoxicity against melanoma cells, phenolic and flavonoid compounds were identified and quantified in *A. distichum* organ extracts using HPLC. Eighteen polyphenolic compounds, including chlorogenic acid, gallic acid, and luteolin, were identified in *A. distichum* organ extract (Table 1). AL (78.054 ± 0.307 mg/g of extract) and AF (36.012 ± 0.392 mg/g of extract) contain gallic acid (Table 1), which induces apoptosis in cancer cells [15]. Additionally, the highest amount of taxifolin, which exhibits promising pharmacological activities, including the management of tumors and liver disorders [16], was detected in AL (16.977 ± 0.167 mg/g of extract). Although AB exhibited a low level of cytotoxic activity compared with other extracts, various polyphenolic compounds, including kaempferol, rhamnetin, tangeretin, vanillic acid, and ferulic acid, have been identified. Taken together, the results show that the anti-melanoma activity of *A. distichum* organ extract should be mediated by these active polyphenolic compounds together with phytochemicals, which were not identified in this study. Additionally, differences in the anti-melanoma activity of organ extracts might be mediated by the different compositions of polyphenolic compounds.

### 2.3. AL Induces Caspase 8-dependent Apoptosis in SK-MEL-2 Cells

**Figure 2:**
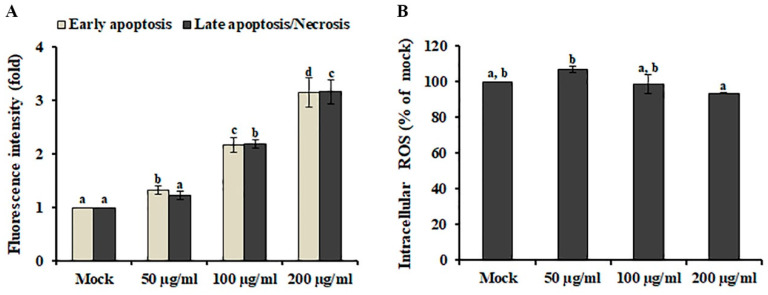
Analysis of *Abeliophyllum distichum* leaves extract (AL)-induced apoptosis and ROS production in SK-MEL-2 cells. (**A**) Early apoptotic cells and dead cells (late-stage apoptotic or necrotic cells) were analyzed using a fluorescence reader after Annexin V and PI staining. (**B**) Intracellular ROS generation was detected in SK-MEL-2 cells after treatment with different concentrations of AL by staining with DCFH-DA. The data are expressed as means ± SE. Values with different superscript letters are significantly different (*p* < 0.05).

Apoptosis and necrosis are highly conserved cell death mechanisms involved in the elimination of cancer cells by anti-cancer agents [17]. Distinguishing specific cell death subtypes is highly important to develop antineoplastic or other therapeutic drugs. To investigate the cell death type induced by AL, early-stage apoptotic cells and dead cells (late-stage apoptotic or necrotic cells) were discriminated using Annexin V/propidium iodide (PI) staining assay combined with fluorescence plate reader measurements. As shown in Figure 2A, early-stage apoptotic cells and dead cells were significantly increased in SK-MEL-2 cells after AL treatment in a dose-dependent manner, suggesting that AL-induced cell death is comparable between early apoptotic and dead cells (late-stage apoptotic or necrotic cells) in SK-MEL-2 cells.

Reactive oxygen species (ROS), generated through various extracellular and intracellular actions, function as very important intracellular messengers involved in the growth, differentiation, progression, metabolism, and death of cells [18]. In cancer cells, low doses of ROS promote cell proliferation in various cancer cell types, although excessive levels of ROS activate several signaling pathways involved in cell apoptosis [19]. Therefore, ROS generation by phytochemicals has been closely associated with the induction of apoptosis in cancers [20]. To investigate whether AL-induced cell death is mediated by ROS accumulation, the intracellular ROS level in AL-treated cells was measured using the fluorogenic probe DCFH-DA. As shown in Figure 2B, AL treatment did not result in the enhancement of intracellular ROS production, indicating that AL is a source of anticancer compounds that will not cause oxidative damage to normal cells.

**Figure 3:**
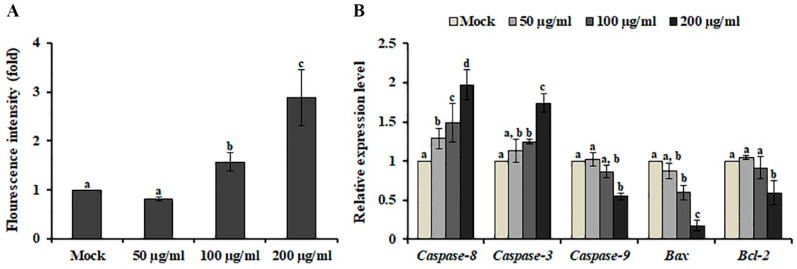
Effect of *Abeliophyllum distichum* leaves extract on caspase-3 activity and gene expression in SK-MEL-2 cells. The activity of caspase-3 (**A**) and expression of genes involved in the extrinsic and intrinsic apoptotic pathways (**B**) were analyzed after AL treatment for 48 h. The transcript level of each gene was normalized to the constitutive expression level of *GAPDH* and was expressed relative to the values of the mock-treated control. Data represent the means ± SE of three independent experiments. Different superscript letters represent significant differences compared with the mock-treated control (*p* < 0.05).

Caspase-3, an intracellular cysteine protease, is the main executioner of apoptosis [21]. When SK-MEL-2 cells were treated with AL, caspase-3 activity was increased in a dose-dependent manner (Figure 3A), indicating that AL suppressed the viability of SK-MEL-2 cells and led to programmed cell death through the modulation of caspase-3 activity. Caspase-3 activation is induced by caspase-8 and caspase-9, which are involved in the extrinsic and intrinsic apoptotic pathways, respectively [22]. To determine the underlying mechanism of AL-induced cell death, we analyzed the gene expression level of *caspase-8* and *caspase-9* in AL-treated SK-MEL-2 cells. An increased level of *caspase-8* and *caspase-3* expression was observed after AL treatment, whereas *caspase-9* expression was decreased (Figure 3B). Additionally, down-regulation of the *Bax*/*Bcl-2* ratio, which regulates activation of the intrinsic apoptotic pathway [23], was observed after AL treatment, indicating that the extrinsic pathway triggers apoptosis in response to AL.

### 2.4. AL Induces Cell Death via the MEK-ERK Independent Pathway

**Figure 4:**
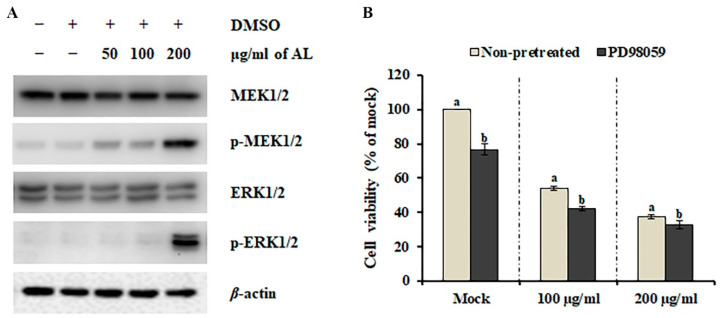
Effect of the MEK/ERK signaling pathway on *Abeliophyllum distichum* leaves extract (AL)-induced cell death. (**A**) The protein and activation levels of MEK1/2 and ERK1/2 were analyzed using western blotting after AL treatment. (**B**) Cell viability of SK-MEL-2 cells co-treated with AL and PD98059 for 48 h was assessed using MTT assay. Values in the same column with different superscript letters are significantly different (*p* < 0.05).

The mitogen-activated protein kinase (MAPK) pathway is essential in regulating cellular processes, including proliferation, differentiation, development, survival, and, under some conditions, also apoptosis [24]. Therefore, the involvement of MAPKs has been highlighted recently in various cell death-inducing models. To investigate the involvement of the MAPK pathway in AL-induced cell death, we analyzed the phosphorylation (activation) status of major MAPKs, MEK1/2 and ERK1/2. As shown in Figure 4A, dynamic activation of MEK1/2 and ERK 1/2 was observed after AL treatment. Although several phytochemicals that can inhibit the MEK-ERK pathway have been suggested as potential cancer drugs [25], MEK-ERK-dependent multiple caspase activation is essential for mouse fibroblast and glioma cell death [26,27]. Therefore, we presumed that activation of the MEK-ERK pathway is involved in AL-induced cell death. To test this hypothesis, SK-MEL-2 cells were co-treated with AL (100 μg/ml and 200 μg/ml) and the MEK1/2 selective inhibitor PD98059 for 48 h, and cell death was determined using MTT assay. PD98059 did not inhibit AL-induced cell death (Figure 4B), suggesting that activation of the MEK-ERK pathway is not essential for the anticancer activity of AL against SK-MEL-2 cells.

### 2.5. AL Down-Regulates the Oncogenic Phosphoinositide 3-kinase (PI3K)/Akt Pathway in SK-MEL-2 Cells

DEG analysis using RNA-seq is a powerful method that can explain the biological changes during the development of a new drug and describe the molecular mechanism of new agents according to the gene expression pattern [28]. To obtain a global overview of the molecular mechanisms involved in AL-induced cell death, total RNA was extracted from mock- or AL-treated cells and was used to generate transcriptome libraries on an Illumina HiSeq™ 2500 sequencing platform. After removing low-quality reads, 67 to 78 million clear reads (9.8 to 11.5 Gb) from each sample were acquired for DEGs analysis. By employing an RPKM (reads per kilobase per million mapped reads) cut-off of 0.05 to identify DEGs, 710 DEGs (182 up-regulated and 528 down-regulated) were detected comparing the mock- and AL-treated libraries (Table S1). To understand the universal response of SK-MEL-2 cells to 200 μg/ml of AL, all screened DEGs were subjected to gene ontology (GO) enrichment analysis, resulting in 54 functional groups classified into three categories: “cellular component”, “molecular function”, and “biological process” (Figure S1). The category “biological process”, comprising 27 functional groups, exhibited the highest number of annotations alongside “cellular process”, followed by “biological regulation”, “metabolic process”, and “regulation of biological process”. Genes within the same pathway usually cooperate to exercise their biological function [29], suggesting that pathway enrichment is a useful strategy to help shed light on the biological mechanism. Pathway enrichment analysis of the identified DEGs revealed that DEGs were mostly enriched in the PI3K/Akt signaling pathway and Rap1 signaling pathway in SK-MEL-2 cells (Figure S2A). Among DEGs, nine genes, PDGFC, IGF1, KDR, IGF1R, PIK3R1, ITGB3, PDGFD, Akt3, and LPAR3, were involved in both the PI3K/Akt signaling pathway and Rap1 signaling pathway (Figure S2B), supporting that PI3K and Akt serve as downstream signaling molecules of Rap1 [30]. To validate the gene expression results from RNA-seq, the *Akt 3* expression (Figure S3A) and protein levels (Figure S3B) of Akt3 were analyzed using qRT-PCR and western blotting. As expected, *Akt3* showed a consistent expression pattern with its profile revealed using RNA-Seq (Figure S3A). The PI3K/Akt pathway signaling plays a significant role in the prevention of apoptosis by inhibiting caspase-dependent apoptosis [31,32]. In SK-MEL-2 cells, most of the DEGs involved in the PI3K/Akt pathway were down-regulated by AL treatment (Table 2), suggesting that AL-induced caspase-8 and -3 might be mediated by down-regulation of the PI3K/Akt pathway.

## 3. Discussion

Although cancer therapies have been performed throughout history, the efficacy and safety of treatment remain a challenge. Thus, medicinal plants and phytochemicals have been suggested as complementary treatments and primary sources of synthetic drugs. In this study, we have determined the anti-melanoma activity of *A. distichum* organ extracts and suggest that AL is a potent source of selective anticancer agents (Figure 1). The difference in the anti-melanoma activity found here between different tested extracts should be due to the difference in the composition of phytochemicals, including polyphenolic compounds (Table 1). One possible explanation for the difference in the composition of polyphenolic compounds is the organ-specific expression of genes associated with polyphenolic compounds. The organ-specific expression pattern of anthocyanin biosynthesis-related genes affects the organ-specific accumulation of metabolites in *A. distichum* [33]. Additionally, other factors, such as catabolism, translocation, and feedback regulation, play important roles in the organ-specific accumulation of metabolites [34].

**Figure 5:**
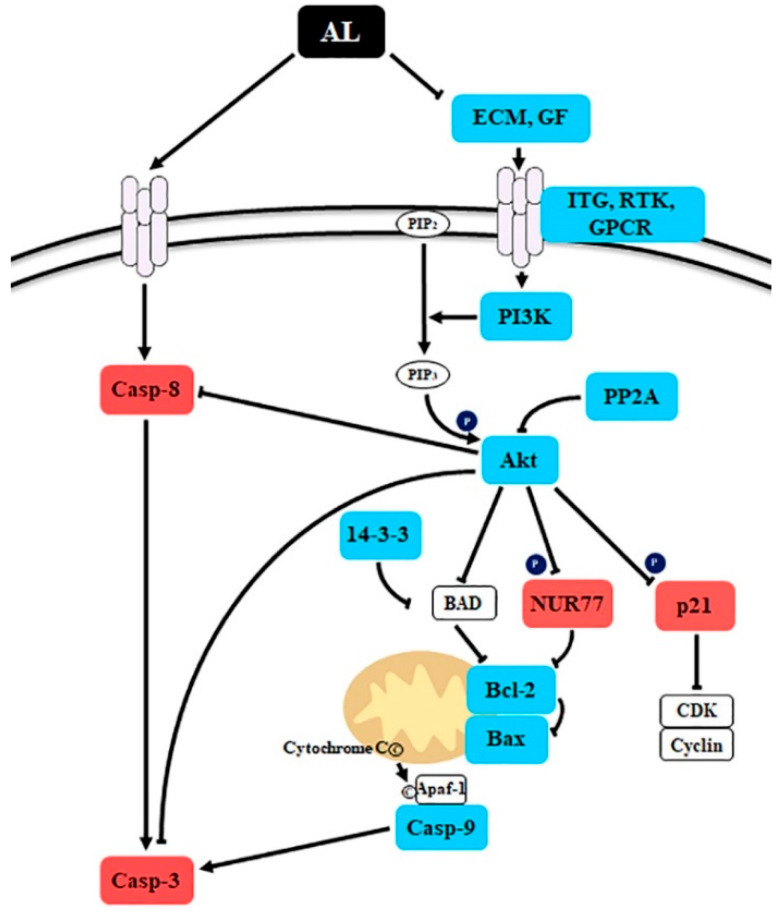
Schematic presentation of the probable molecular mechanism of *Abeliophyllum distichum* leaves extract (AL)-induced cell death. The red and blue boxes indicate the up- and down-regulated genes by AL treatment in SK-MEL-2 cells.

Caspases are a family of cysteine proteases that are responsible for apoptosis initiation and execution and can be activated via two main apoptotic signaling mechanisms: the intrinsic (mitochondrial) pathway and extrinsic (receptor-mediated) pathway [35]. In AL-treated SK-MEL-2 cells, we observed the enhanced activity of caspase-3 (Figure 3A), which converges both apoptotic signaling pathways [21]. However, gene expression analysis indicated that AL induces cell death via activation of the extrinsic pathway (Figure 3B). In human colorectal cancer cells, the ethyl acetate fraction of 80% methanol extract obtained from the plant parts of *A. distichum* including the flower, leaf, and branch induced transcriptional activation of activating transcription factor 3 (ATF3), resulting in apoptosis induction [12]. Various human cancers, including liver cancer, prostate cancer, colon cancer, and multiple myeloma, exhibit a low level of *ATF3* expression [36,37,38,39]. Additionally, the knockdown mutation of *ATF3* promotes the development of prostate cancer through activation of PI3K/Akt signaling [40], which is crucial for the growth, survival, and development of prostate cancer cells [41] and suggests that ATF3 is a promising cancer preventive-therapeutic target. Similarly, we observed up-regulation of *ATF3* expression (Table S1), but down-regulation of the PI3K/Akt signaling pathway when SK-MEL-2 cells were treated with AL (Table 2). Interestingly, treatment with perifosine, an Akt inhibitor, induces extrinsic caspase-dependent cell death [42]. Additionally, gallic acid, the major compound identified from AL (Table 1), has been shown to inhibit the activation of Akt in OVCAR-3 cancer cells, resulting in the suppression of cell viability [43]. Furthermore, AL contained the highest content of taxifolin (Table 1), which suppressed the PI3K/Akt/mTOR pathway and scar cell carcinoma growth by inducing apoptosis [44]. Taken together, these results indicate that AL-induced cell death is mediated by down-regulation of the PI3K/Akt signaling pathway and activation of caspase 8-dependent apoptosis (Figure 5).

## 4. Conclusions

In conclusion, the results of the present study indicate the anti-melanoma potential of AL. AL positively or negatively regulates the complex network of signaling pathways involved in cell cytotoxicity, indicating that the activity described here may be caused by a combination of several potential anti-melanoma compounds. In addition, further study is necessary to analyze its effect on cancer animal models.

## 5. Materials and Methods

### 5.1. Materials

SK-MEL-2 human melanoma cells (ATCC® HTB-68™), B16F10 murine melanoma cells (ATCC® CRL-6475™), and HDFa human dermal fibroblasts (ATCC® PCS-201-012™) were purchased from ATCC (American Type Culture Collection, Manassas, Virginia, USA). Dimethyl sulfoxide (DMSO), 3-(4,5-dimethylthiazol-2-yl)-2,5-diphenyltetrazolium bromide (MTT), and 2’,7’-dichlorofluorescein diacetate (DCFH-DA) were purchased from Duchefa (Seoul, Korea). RPMI 1640 medium, DMEM medium, penicillin, and fetal bovine serum (FBS) were purchased from Biowest (Riverside, MO, USA). The Pierce™ BCA Protein Assay Kit and SuperSignal™ West Pico PLUS Chemiluminescent Substrate were purchased from Thermo Fisher Scientific (Waltham, Massachusetts, USA). The antibodies for mitogen-activated protein kinase (MEK)1/2, phospho-MEK1/2 (Ser217/221), extracellular signal-regulated kinase (ERK)1/2, and phospho-ERK1/2 (Thr202/Tyr204) were obtained from Cell Signaling Technology (Beverly, Massachusetts, USA). The β-actin antibody was purchased from Santa Cruz Biotechnology, Inc. (Dallas, Texas, USA). The caspase-3 assay kit was purchased from Biovision (Milpitas, California, USA). The Annexin V FITC assay kit was purchased from Cayman Chemical (Ann Arbor, Michigan, USA).

### 5.2. Extraction and Sample Preparation

*A. distichum* fruit, branches, and leaves were grown and taken from the research forest at Chungbuk National University. The fruit, branches, and leaves of *A. distichum* were lyophilized and ground into a fine powder using a blender. The ground materials were soaked in methanol for 24 h and subjected to ultrasonication (1 h × 3 times). After filtration, the methanol extracts of the fruit (AF), branches (AB), and leaves (AL) were evaporated using a rotary vacuum evaporator and kept in a refrigerator until use.

### 5.3. Cell Culture

SK-MEL-2 human melanoma cells, B16F10 murine melanoma cells, and HDFa human dermal fibroblasts were cultured in RPMI 1640 medium supplemented with 10% fetal bovine serum (FBS) and antibiotics or in DMEM medium with 10% FBS and antibiotics, at 37 °C in a humidified chamber containing 5% CO_2_.

### 5.4. Cell Viability Assay

Cell viability was analyzed using MTT assay as described by Jin et al. [45]. Cells were plated in 96-well plates at a density of 2 × 10^4^ cells/well for SK-MEL-2 cells or 1 × 10^4^ cells/well for B16F10 cells or HDFa cells and then were cultured for 24 h. Next, the cells were treated with different concentrations (25 to 400 μg/ml) of each organ extract. After treatment for 48 h (or respective time points), 20 μL of MTT solution (1 mg/ml, diluted with phosphate-buffered saline (PBS)) were added and the cells were incubated in a CO_2_ incubator for an additional 4 h. The produced formazan dissolved with DMSO was quantified at 550 nm using an iMARK™ microplate reader (Bio-Rad, Hercules, CA, USA). An extract amount < 50 μg/ml did not show significant cytotoxic activity, whereas > 200 μg/ml of extracts exhibited similar cytotoxic activity compared to 200 μg/ml of extracts. Based on this finding, three different concentrations (50 μg/ml, 100 μg/ml, and 200 μg/ml) of each organ extract were used for further analysis.

### 5.5. Intracellular Reactive Oxygen Species (ROS) Measurement

Intracellular ROS were detected using the fluorogenic probe DCFH-DA as described by Warleta et al. [46] with minor modifications. SK-MEL-2 cells were exposed to different concentrations (50 μg/ml, 100 μg/ml, and 200 μg/ml) of AL for 5 h and then were stained with 20 µM DCFH-DA. After incubation in a CO_2_ incubator for 30 min, the cells were washed with PBS, and then DCF fluorescence was measured using a SpectraMAX Gemini EM Fluorescence Microplate Reader (Molecular Devices, San Jose, CA, USA) at the excitation and emission wavelengths of 485 nm and 525 nm, respectively.

### 5.6. Annexin V/propidium Iodide (PI) Staining Assay

Early-stage apoptotic cells and dead cells were analyzed using an Annexin V FITC Assay Kit (Cayman Chemical, Ann Arbor, MI, USA) according to the manufacturer’s instructions. Early-stage apoptotic cells stained using Annexin V FITC were detected with excitation and emission wavelengths of 560 nm and 595 nm, respectively, whereas dead cells stained using PI were detected with excitation and emission wavelengths of 485 nm and 535 nm, respectively, using a SpectraMAX Gemini EM Fluorescence Microplate Reader (Molecular Devices, San Jose, CA, USA).

### 5.7. Caspase-3 Activity

Caspase-3 activity was analyzed using a Caspase-3/CPP32 Fluorometric Assay Kit (BioVision, Milpitas, CA, USA) according to the manufacturer’s instructions. Fifty micrograms of protein obtained from mock- or AL-treated cells were mixed with each substrate and then incubated at 37 °C for 2 h. Caspase-3 activity was measured using a fluorescence microplate reader equipped with a 400-nm excitation filter and a 505-nm emission filter.

### 5.8. Western Blotting

Proteins were extracted from mock- or AL-treated cells using RIPA buffer (50 mM Tris-HCl (pH 7.5), 150 mM NaCl, 1% Triton X-100, 0.1% sodium dodecyl sulfate (SDS), 0.5% sodium deoxycholate, 1 mM ethylenediaminetetraacetic acid, and 10 mM NaF). The Pierce™ BCA Protein Assay Kit was used to quantify the proteins according to the manufacturer’s instructions. Ten micrograms of protein were separated using 10% SDS-polyacrylamide gel electrophoresis (10% gel) and were transferred to a polyvinylidene fluoride membrane. After blocking using 5% nonfat dried milk, the membranes were incubated overnight at 4 °C with antibodies against MEK1/2, p-MEK1/2, ERK1/2, p-ERK1/2, and β-actin. The protein bands were subsequently visualized using ECL reagent (SuperSignal™ West Pico PLUS Chemiluminescent Substrate; Thermo Fisher Scientific, Carlsbad, CA, USA) and an Azure c280 imager (Azure Biosystems, Inc., Dublin, CA, USA).

### 5.9. RNA Sequencing and DEG Analysis

Total RNA extracted from mock- or AL-treated cells was quantified using a DeNovix DS-11 (DeNovix Inc, Wilmington, DE, USA). The cDNA library was synthesized and sequenced using an Illumina HiSeq™ 2500 sequencing platform. Adapter sequences, empty reads, low-quality reads (with ambiguous sequence, N), and reads with more than 10% Q < 20 bases (i.e., with a base quality less than 20) were discarded from the raw sequencing data using the FastQC and Trimmomatic tools. Next, the clean reads were mapped to the *Homo sapiens* genome reference (*Homo sapiens* genome assembly GRCh38.p13) using an HISAT2 aligner. Transcript levels were calculated using SAMtools, and relative transcript abundances were analyzed using DEseq [47]. Statistically significant DEGs (*p* value cutoff of 0.05 and adjusting to |log2 (fold change) |≥ 1) were analyzed for gene ontology (GO) enrichment and pathway enrichment using the Blast2GO program and KOBAS v.3.0 Server, respectively. The statistical significance threshold level for all GO enrichment and pathway enrichment was *p* < 0.05. All reads were deposited in the National Agricultural Biotechnology Information Center (NABIC, http://nabic.rda.go.kr) with accession number NN-6325 and NN-6326.

### 5.10. Quantitative Real-Time Polymerase Chain Reaction (qRT–PCR)

One hundred nanograms of total RNA were reverse transcribed into cDNA using a ReverTra Ace® qPCR RT Master Mix with qDNA Remover (TOYOBO Co., Ltd, Osaka, Japan) according to the manufacturer’s instructions. qRT–PCR was performed using SYBR® Green Real-time PCR Master Mix (TOYOBO, Co., Ltd, Osaka, Japan) in a CFX96TM Real-Time System (Bio-Rad, Hercules, CA, USA). The relative expression levels of selected genes were normalized to the expression of the internal reference gene GAPDH. The specific primer pairs are listed in Table S2.

### 5.11. HPLC Analysis

HPLC analysis was performed using a Shimadzu liquid chromatography system (LC-10ADvp) coupled with an ultraviolet-visible detector (SPD-10A; Shimadzu, Japan). Separation was achieved using a Luna® HPLC column (C18(2) 100 Å, 250 × 4.6 mm, 5 μm) at 35 °C with a 0.7-ml/min flow rate. The mobile phases consisted of water containing 0.1% trifluoroacetic acid (mobile phase A) and acetonitrile containing 0.1% trifluoroacetic acid (mobile phase B). The following gradient elution was performed: 10% B in 0–0.01 min, 10–40% B in 0.01−28 min, 40–60% B in 28–39 min, 60–90% B in 39–50 min, and holding at 90% B for 5 min. The concentration was calculated by comparing the peak areas of the samples with the calibration curve of the standards.

### 5.12. Statistical Analysis

All experiments were performed for three independent replicates, and the data were expressed as means ± standard error (SE). The significance of the between-group differences was determined using analysis of Duncan’s multiple range test. Values of *p* < 0.05 were considered significant.

## Figures and Tables

**Table 1:** Polyphenolic compounds in *Abeliophyllum distichum* organ extracts.

	Fruit	Stem	Leaf
Rutin	6.966 ± 0.073 ^c,1^	7.630 ± 0.150 ^b^	8.768 ± 0.013 ^a^
Taxifolin	1.483 ± 0.019 ^c^	3.556 ± 0.027 ^b^	16.977 ± 0.167 ^a^
Narirutin	5.110 ± 0.056 ^b^	1.447 ± 0.038 ^c^	9.727 ± 0.200 ^a^
Naringin	3.715 ± 0.016 ^a^	ND	ND
Neohesperidin	ND	ND	0.401 ± 1.626 ^a^
Myricetin	8.987 ± 0.329 ^b^	9.177 ± 0.204 ^a^	0.816 ± 0.079 ^c^
Quercetin	ND	4.758 ± 0.185 ^a^	ND
Luteolin	3.220 ± 0.027 ^b^	252.818 ± 1.261 ^a^	1.442 ± 0.024 ^c^
Naringenin	ND	2.923 ± 0.575 ^a^	0.786 ± 1.391 ^b^
Kaempferol	ND	0.360 ± 0.014 ^a^	ND
Isorhamnetin	ND	7.383 ± 0.151 ^a^	7.041 ± 0.121 ^b^
Rhamnetin	ND	2.131 ± 0.019 ^a^	ND
Tangeretin	ND	5.068 ± 0.040 ^a^	ND
Gallic acid	36.012 ± 0.392 ^b^	3.099 ± 0.042 ^c^	78.054 ± 0.307 ^a^
Vanillic acid	ND	0.217 ± 0.024 ^a^	ND
Chlorogenic acid	0.605 ± 0.095 ^b^	ND	3.960 ± 0.038 ^a^
Ferulic acid	ND	1.144 ± 0.305 ^a^	ND
Sinapinic acid	ND	16.093 ± 0.143 ^a^	1.001 ± 0.196 ^b^

^1^ mg/g of extract values are the average of triplicate experiments. Values with different superscript letters are significantly different between organ extracts (*p* < 0.05). ND, not detected.

**Table 2:** *Abeliophyllum distichum* leaves extract induces differentially expressed genes involved in the phosphoinositide 3-kinase (PI3K)/Akt signaling pathway.

Up/Down	Gene	Gene Description	log2 (Fold Change)	*p*-Value
Up	IL6R	interleukin 6 receptor	2.65	0.0005
	CDKN1A	cyclin dependent kinase inhibitor 1A	1.82	0.0049
	NR4A1	nuclear receptor subfamily 4 group A member 1	1.36	0.0282
Down	CASP9	caspase-9 isoform alpha precursor	−1.09	0.0023
	BCL2	apoptosis regulator Bcl-2 isoform alpha	−1.27	0.0183
	PDGFC	platelet derived growth factor C	−1.29	0.0366
	IGF1	insulin like growth factor 1	−1.32	0.0349
	IGF1R	insulin like growth factor 1 receptor	−1.33	0.0429
	ITGA2	integrin subunit alpha 2	−1.34	0.0386
	TNN	tenascin N	−1.40	0.0325
	LAMA4	laminin subunit alpha 4	−1.36	0.0304
	PIK3R1	phosphoinositide-3-kinase regulatory subunit 1	−1.45	0.0186
	ITGB3	integrin subunit beta 3	−1.46	0.0174
	TNC	tenascin C	−1.68	0.0355
	LPAR3	lysophosphatidic acid receptor 3	−1.76	0.0154
	PDGFD	platelet derived growth factor D	−1.94	0.0212
	PPP2R2B	serine/threonine-protein phosphatase 2A regulatory subunit B	−2.14	0.0051
	Akt3	AKT serine/threonine kinase 3	−2.18	0.0102
	ITGB8	integrin subunit beta 8	−2.22	0.0005
	THBS2	thrombospondin 2	−2.27	0.0265
	KDR	kinase insert domain receptor	−2.70	0.0028
	YWHAE	tyrosine 3-monooxygenase/tryptophan 5-monooxygenase activation protein epsilon	−3.85	0.00002

## Supplementary Materials

The following are available online at https://www.mdpi.com/2223-7747/9/6/757/s1, Table S1. *Abeliophyllum distichum* leaves extract-induced DEGs, Table S2. Primer sequences for real-time PCR analysis, Figure S1. Gene ontology classification of *Abeliophyllum distichum* leaves extract-induced DEGs. Figure S2. KEGG pathway enrichment analysis using *Abeliophyllum distichum* leaves extract DEGs (A). Venn diagram analysis of DEGs involved in PI3K/Akt signaling pathway and Rap1 signaling pathway (B). Figure S3. The expression (A) and protein levels (B) of Akt3 in AL-treated SK-MEL-2 cells.

## References

[1] Kappelmann-Fenzl M., Gebhard C., Matthies A.O., Kuphal S., Rehli M., Bosserhoff A.K. (2019). C-Jun drives melanoma progression in PTEN wild type melanoma cells. Cell Death Dis..

[2] Albuquerque K.R.S., Pacheco N.M., del Rosario Loyo Casao T., de Melo F.C.S.A., Novaes R.D., Gonçalves R.V. (2018). Applicability of plant extracts in preclinical studies of melanoma: A systematic review. Mediat. Inflamm..

[3] Parsa N. (2012). Environmental factors inducing human cancers. Iran. J. Public Health.

[4] Sample A., He Y.Y. (2018). Mechanisms and prevention of UV-induced melanoma. Photodermatol. Photoimmunol. Photomed..

[5] Chinembiri T.N., Du Plessis L.H., Gerber M., Hamman J.H., Du Plessis J. (2014). Review of natural compounds for potential skin cancer treatment. Molecules.

[6] Choi J.H., Seo E.J., Sung J., Choi K.M., Kim H., Kim J.S., Lee J., Efferth T., Hyun T.K. (2017). Polyphenolic compounds, antioxidant and anti-inflammatory effects of Abeliophyllum distichum Nakai extract. J. Appl. Bot. Food Qual..

[7] Lee J., Kang Y. (2018). Anti-inflammatory Effects of Abeliophyllum distichum Flower Extract and Associated MAPKs and NF- κ B Pathway in Raw264. 7 Cells. Korean J. Plant Resour..

[8] Jang T., Park J.H. (2018). Antioxidant activity and inhibitory effects on oxidative DNA damage of callus from Abeliophyllum distichum nakai. Korean J. Plant Resour..

[9] Jang T., Choi J., Kim H., Lee E., Han M., Lee K., Kim D., Jang T., Choi J., Kim H. (2018). Whitening Activity of Abeliophyllum distichum Nakai Leaves According to the Ratio of Prethanol A in the Extracts. Korean J. Plant Resour..

[10] Li H.M., Kim J.K., Jang J.M., Cui C.B., Lim S.S. (2013). Analysis of the inhibitory activity of Abeliophyllum distichum leaf constituents against aldose reductase by using high-speed counter current chromatography. Arch. Pharm. Res..

[11] Oh H., Kang D.G., Kwon T.O., Jang K.K., Chai K.Y., Yun Y.G., Chung H.T., Lee H.S. (2003). Four glycosides from the leaves of Abeliophyllum distichum with inhibitory effects on angiotensin converting enzyme. Phyther. Res..

[12] Park H.H., Park H.H., Eo J.J., Song M.M., Woo H.H., Kim K.K., Lee W.W., Lee H.H., Lee R.R., Koo S.S. (2014). The induction of activating transcription factor 3 (ATF3) contributes to anti-cancer activity of Abeliophyllum distichum Nakai in human colorectal cancer cells. BMC Complement Altern. Med..

[13] Iqbal J., Abbasi B.A., Mahmood T., Kanwal S., Ali B., Shah S.A., Khalil A.T. (2017). Plant-derived anticancer agents: A green anticancer approach. Asian Pac. J. Trop. Biomed..

[14] Sajadimajd S., Bahramsoltani R., Iranpanah A., Kumar Patra J., Das G., Gouda S., Rahimi R., Rezaeiamiri E., Cao H., Giampieri F. (2019). Advances on Natural Polyphenols as Anticancer Agents for Skin Cancer. Pharmacol. Res..

[15] Zhao B., Hu M. (2013). Gallic acid reduces cell viability, proliferation, invasion and angiogenesis in human cervical cancer cells. Oncol. Lett..

[16] Sunil C., Xu B. (2019). An insight into the health-promoting effects of taxifolin (dihydroquercetin). Phytochemistry.

[17] Lekshmi A., Varadarajan S.N., Lupitha S.S., Indira D., Ann Mathew K., Nair A.C., Nair M., Prasad T., Sekar H., Gopalakrishnan A.K. (2017). A quantitative real-time approach for discriminating apoptosis and necrosis. Cell Death Discov..

[18] Zaidieh T., Smith J.R., Ball K.E., An Q. (2019). ROS as a novel indicator to predict anticancer drug efficacy. BMC Cancer.

[19] Mitra S., Nguyen L.N., Akter M., Park G., Choi E.H., Kaushik N.K. (2019). Impact of ROS generated by chemical, physical, and plasma techniques on cancer attenuation. Cancers.

[20] Jeong C.-H., Joo S.H. (2016). Downregulation of Reactive Oxygen Species in Apoptosis. J. Cancer Prev..

[21] Lossi L., Castagna C., Merighi A. (2018). Caspase-3 mediated cell death in the normal development of the mammalian cerebellum. Int. J. Mol. Sci..

[22] Wu M.H., Jin X.K., Yu A.Q., Zhu Y.T., Li D., Li W.W., Wang Q. (2014). Caspase-mediated apoptosis in crustaceans: Cloning and functional characterization of EsCaspase-3-like protein from Eriocheir sinensis. Fish. Shellfish Immunol..

[23] Campbell K.J., Tait S.W.G. (2018). Targeting BCL-2 regulated apoptosis in cancer. Open Biol..

[24] Shaul Y.D., Seger R. (2007). The MEK/ERK cascade: From signaling specificity to diverse functions. Biochim. Biophys. Acta Mol. Cell Res..

[25] Braicu C., Buse M., Busuioc C., Drula R., Gulei D., Raduly L., Rusu A., Irimie A., Atanasov A.G., Slaby O. (2019). A comprehensive review on MAPK: A promising therapeutic target in cancer. Cancers.

[26] Stefanelli C., Tantini B., Fattori M., Stanic’ I., Pignatti C., Clo C., Guarnieri C., Caldarera C.M., Mackintosh C.A., Pegg A.E. (2002). Caspase activation in etoposide-treated fibroblasts is correlated to ERK phosphorylation and both events are blocked by polyamine depletion. FEBS Lett..

[27] Tomiyama A., Tachibana K., Suzuki K., Seino S., Sunayama J., Matsuda K.I., Sato A., Matsumoto Y., Nomiya T., Nemoto K. (2010). MEK-ERK-dependent multiple caspase activation by mitochondrial proapoptotic Bcl-2 family proteins is essential for heavy ion irradiation-induced glioma cell death. Cell Death Dis..

[28] Chu Y., Corey D.R. (2012). RNA sequencing: Platform selection, experimental design, and data interpretation. Nucleic Acid Ther..

[29] Wenping H., Yuan Z., Jie S., Lijun Z., Zhezhi W. (2011). De novo transcriptome sequencing in Salvia miltiorrhiza to identify genes involved in the biosynthesis of active ingredients. Genomics.

[30] Park J.Y., Juhnn Y.S. (2017). CAMP signaling increases histone deacetylase 8 expression via the Epac2-Rap1A-Akt pathway in H1299 lung cancer cells. Exp. Mol. Med..

[31] Jeong S.J., Dasgupta A., Jung K.J., Um J.H., Burke A., Park H.U., Brady J.N. (2008). PI3K/AKT inhibition induces caspase-dependent apoptosis in HTLV-1-transformed cells. Virology.

[32] Chang F., Lee J.T., Navolanic P.M., Steelman L.S., Shelton J.G., Blalock W.L., Franklin R.A., McCubrey J.A. (2003). Involvement of PI3K/Akt pathway in cell cycle progression, apoptosis, and neoplastic transformation: A target for cancer chemotherapy. Leukemia.

[33] Choi J.H., Kim H., Hyun T.K. (2018). Transcriptome analysis of Abeliophyllum distichum NAKAI reveals potential molecular markers and candidate genes involved in anthocyanin biosynthesis pathway. S. Afr. J. Bot..

[34] Matsuda F., Hirai M.Y., Sasaki E., Akiyama K., Yonekura-Sakakibara K., Provart N.J., Sakurai T., Shimada Y., Saito K. (2010). AtMetExpress development: A phytochemical atlas of Arabidopsis development. Plant Physiol..

[35] Nair P., Lu M., Petersen S., Ashkenazi A. (2014). Apoptosis initiation through the cell-extrinsic pathway. Methods Enzymol..

[36] Xiaoyan L., Shengbing Z., Yu Z., Lin Z., Chengjie L., Jingfeng L., Aimin H. (2014). Low expression of activating transcription factor 3 in human hepatocellular carcinoma and its clinicopathological significance. Pathol. Res. Pract..

[37] Ri M. (2016). Endoplasmic-reticulum stress pathway-associated mechanisms of action of proteasome inhibitors in multiple myeloma. Int. J. Hematol..

[38] Wang Z., Yan C. (2016). Emerging roles of ATF3 in the suppression of prostate cancer. Mol. Cell. Oncol..

[39] Park G.H., Song H.M., Jeong J.B. (2017). Kahweol from coffee induces apoptosis by upregulating activating transcription factor 3 in human colorectal cancer cells. Biomol. Ther..

[40] Wang Z., Xu D., Ding H.F., Kim J., Zhang J., Hai T., Yan C. (2015). Loss of ATF3 promotes Akt activation and prostate cancer development in a Pten knockout mouse model. Oncogene.

[41] Song M.S., Salmena L., Pandolfi P.P. (2012). The functions and regulation of the PTEN tumour suppressor. Nat. Rev. Mol. Cell Biol..

[42] Chiarini F., Del Sole M., Mongiorgi S., Gaboardi G.C., Cappellini A., Mantovani I., Follo M.Y., McCubrey J.A., Martelli A.M. (2008). The novel Akt inhibitor, perifosine, induces caspase-dependent apoptosis and downregulates P-glycoprotein expression in multidrug-resistant human T-acute leukemia cells by a JNK-dependent mechanism. Leukemia.

[43] He Z., Chen A.Y., Rojanasakul Y., Rankin G.O., Chen Y.C. (2016). Gallic acid, a phenolic compound, exerts anti-angiogenic effects via the PTEN/AKT/HIF-1α/VEGF signaling pathway in ovarian cancer cells. Oncol. Rep..

[44] Zhou W., Liu Z., Wang M., Chen D., Zhou L., Guo L. (2019). Taxifolin inhibits the development of scar cell carcinoma by inducing apoptosis, cell cycle arrest, and suppression of PI3K/ AKT/mTOR pathway. J. Buon.

[45] Jin S., Eom S.H., Kim J.S., Jo I.H., Hyun T.K. (2019). Influence of ripening stages on phytochemical composition and bioavailability of ginseng berry (Panax ginsxeng C.A. Meyer). J. Appl. Bot. Food Qual..

[46] Warleta F., Quesada C.S., Campos M., Allouche Y., Beltrán G., Gaforio J.J. (2011). Hydroxytyrosol protects against oxidative DNA damage in human breast cells. Nutrients.

[47] Anders S., Huber W. (2010). Differential expression analysis for sequence count data. Genome Biol..

