# Supplementary material for: Polyphenolic Composition and Anti-Melanoma Activity of White Forsythia (Abeliophyllum distichum Nakai) Organ Extracts
Source: Plants (Basel). 2020 Jun 17;9(6):757. doi: 10.3390/plants9060757 (PMC7356668; doi:10.3390/plants9060757)
Supplement: Supplementary file 1 [file plants-09-00757-s001.pdf]

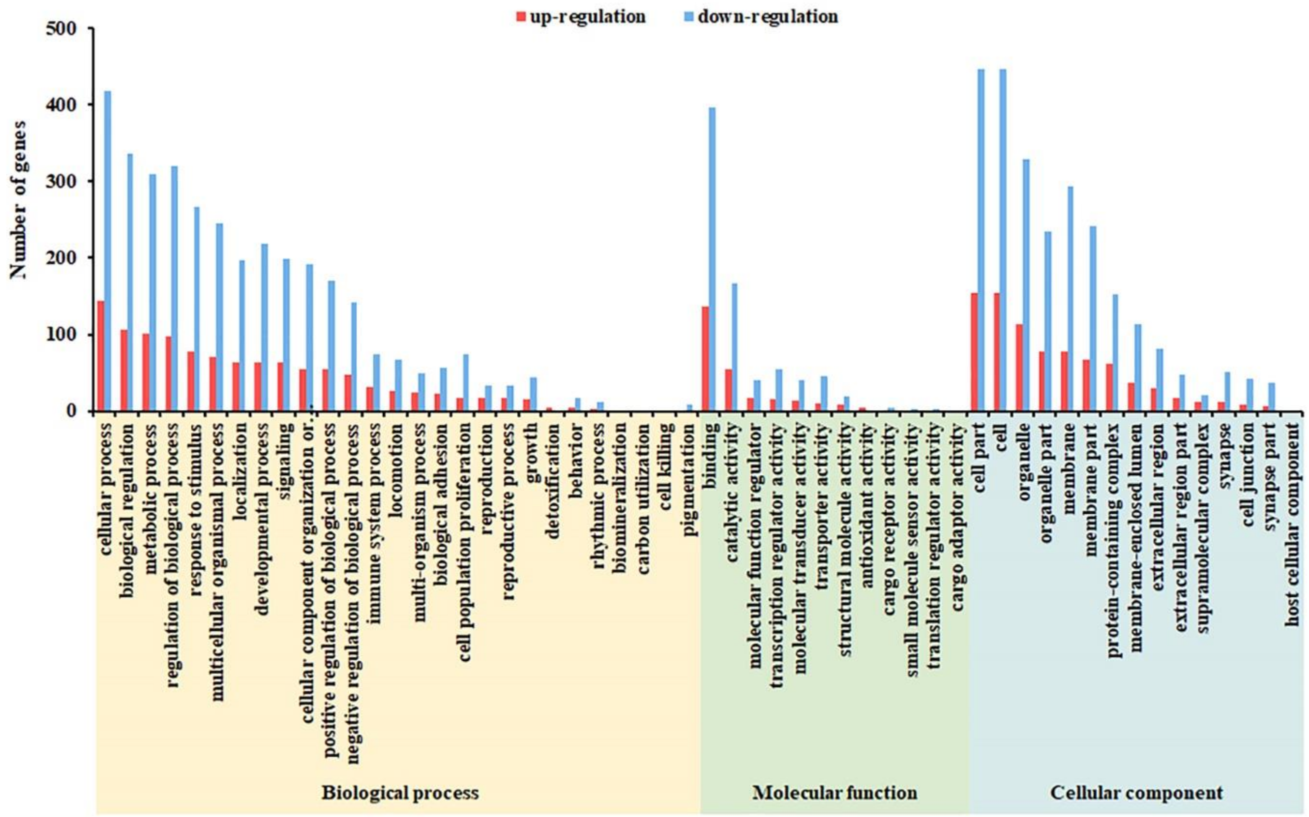

Figure S1. Gene ontology classification of *Abeliophyllum distichum* leaves extract-induced DEGs. The results are summarized in three main categories: Biological process, Cellular component and Molecular function.

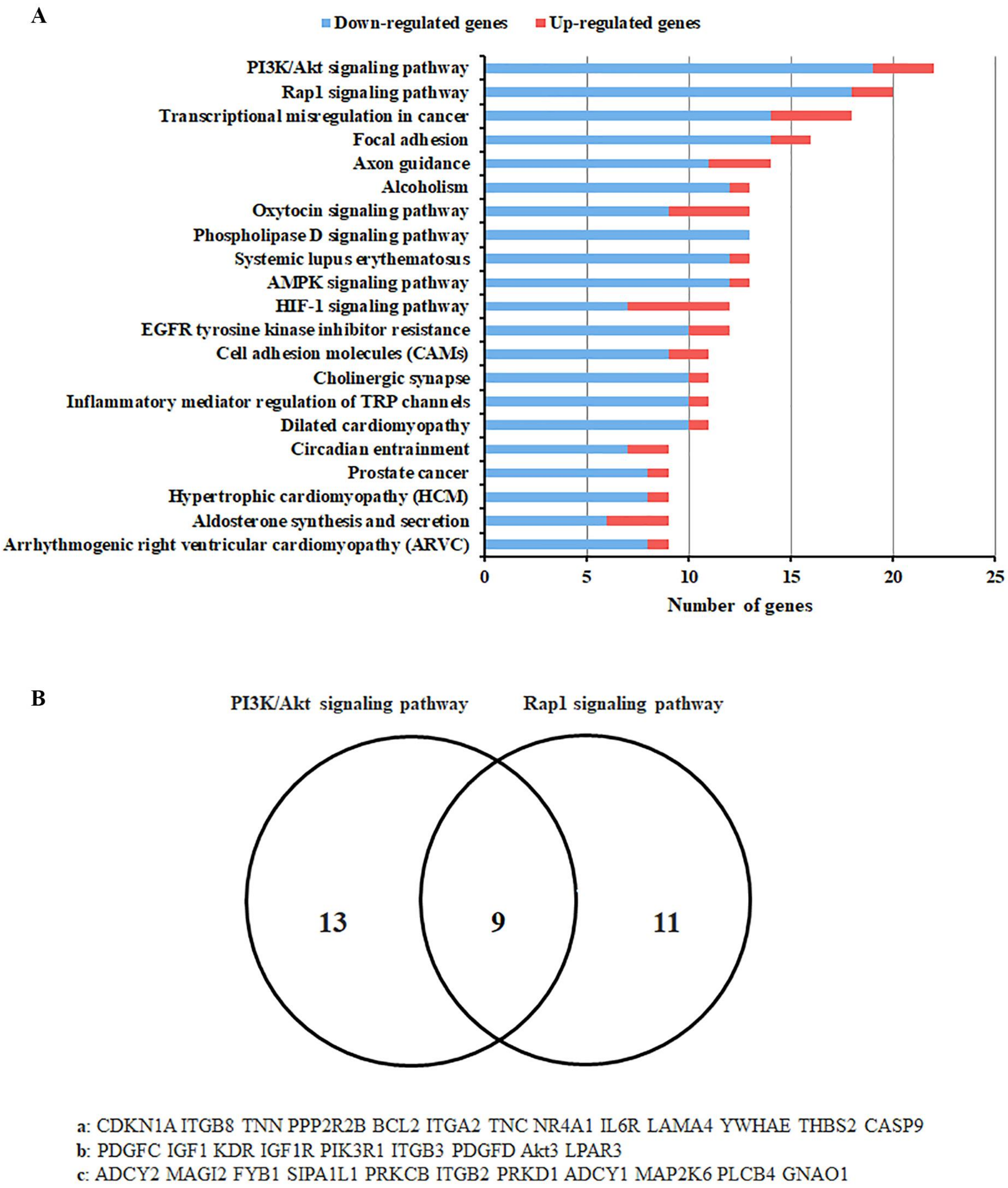

Figure S2. KEGG pathway enrichment analysis using *Abeliophyllum distichum* leaves extract-DEGs (**A**). Venn diagram analysis of DEGs involved in PI3K/Akt signaling pathway and Rap1 signaling pathway (**B**).

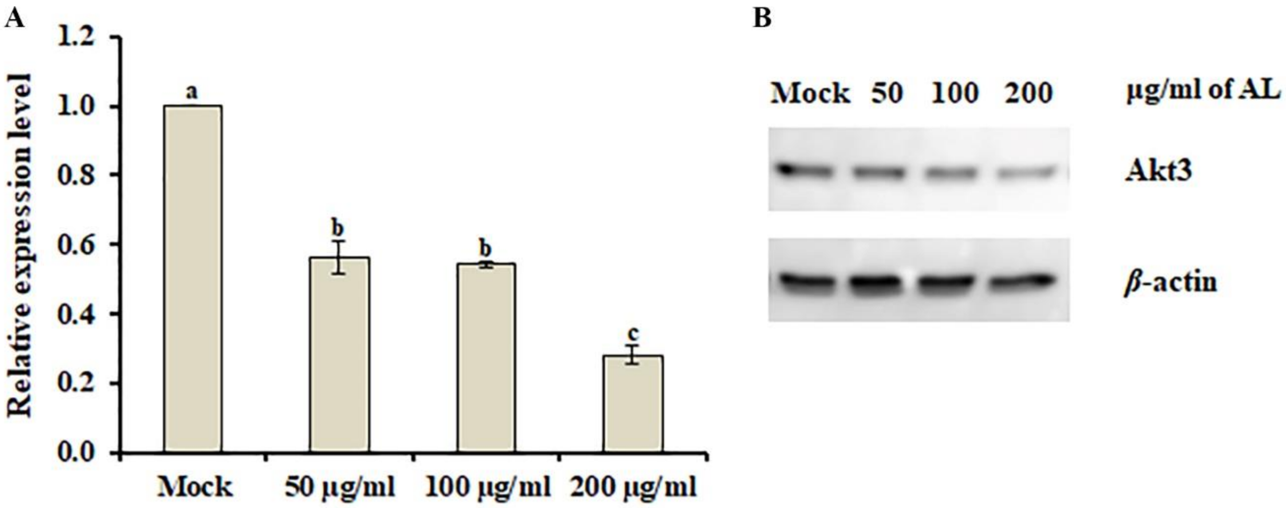

Figure S3. The expression (**A**) and protein levels (**B**) of Akt3 in AL-treated SK-MEL2 cells. Values with different superscripted letters are significantly different ($p < 0.05$).

Table S1. *Abeliophyllum distichum* leaves extract-induced DEGs.

Gene name	Gene description	log2 Fold Change
A2ML1	alpha-2-macroglobulin-like protein 1 isoform 2 [Homo sapiens]	3.45
A4GALT	lactosylceramide 4-alpha-galactosyltransferase [Homo sapiens]	−1.64
ABCB4	phosphatidylcholine translocator ABCB4 isoform A [Homo sapiens]	−1.43
ABCB5	ATP-binding cassette sub-family B member 5 isoform 1 [Homo sapiens]	−2.99
ABHD17C	alpha/beta hydrolase domain-containing protein 17C [Homo sapiens]	−1.62
ABLIM2	actin-binding LIM protein 2 isoform 1 [Homo sapiens]	−2.53
ABTB2	ankyrin repeat and BTB/POZ domain-containing protein 2 [Homo sapiens]	−1.48
ACACA	acetyl-CoA carboxylase 1 isoform 1 [Homo sapiens]	−1.76
ACACB	acetyl-CoA carboxylase 2 precursor [Homo sapiens]	−2.03
ACSM1	acyl-coenzyme A synthetase ACSM1, mitochondrial [Homo sapiens]	−3.05
ADAM19	disintegrin and metalloproteinase domain-containing protein 19 preproprotein [Homo sapiens]	−1.65
ADAM20	disintegrin and metalloproteinase domain-containing protein 20 preproprotein [Homo sapiens]	1.80
ADAM21	disintegrin and metalloproteinase domain-containing protein 21 preproprotein [Homo sapiens]	2.20
ADAMTS6	A disintegrin and metalloproteinase with thrombospondin motifs 6 preproprotein [Homo sapiens]	−1.87
ADARB1	double-stranded RNA-specific editase 1 isoform 1 [Homo sapiens]	−1.39
ADCY1	adenylate cyclase type 1 isoform 2 [Homo sapiens]	−3.08
ADCY2	adenylate cyclase type 2 [Homo sapiens]	−1.85
ADD3	gamma-adducin isoform b [Homo sapiens]	−1.21
ADGRG1	adhesion G-protein coupled receptor G1 isoform b precursor [Homo sapiens]	−1.63
ADGRV1	adhesion G-protein coupled receptor V1 precursor [Homo sapiens]	1.94
AGAP1	arf-GAP with GTPase, ANK repeat and PH domain-containing protein 1 isoform 1 [Homo sapiens]	−1.32
AHSA1	activator of 90 kDa heat shock protein ATPase homolog 1 isoform 2 [Homo sapiens]	1.23
AIG1	androgen-induced gene 1 protein isoform b [Homo sapiens]	−1.41
AK4	adenylate kinase 4, mitochondrial isoform a [Homo sapiens]	−1.37
AKAP8L	A-kinase anchor protein 8-like isoform 2 [Homo sapiens]	1.25
AKT3	RAC-gamma serine/threonine-protein kinase isoform 2 [Homo sapiens]	−2.18
ALDH1L1	cytosolic 10-formyltetrahydrofolate dehydrogenase isoform 1 [Homo sapiens]	1.22
AMOTL2	angiomotin-like protein 2 isoform 1 [Homo sapiens]	1.46
ANAPC11	anaphase-promoting complex subunit 11 isoform 1 [Homo sapiens]	1.51
ANKRD1	ankyrin repeat domain-containing protein 1 [Homo sapiens]	1.97
ANTXR1	anthrax toxin receptor 1 isoform 3 precursor [Homo sapiens]	−1.75
AOC1	amiloride-sensitive amine oxidase [copper-containing] isoform 2 precursor [Homo sapiens]	−3.87
AP2A2	AP-2 complex subunit alpha-2 isoform 1 [Homo sapiens]	−2.12
APOBEC3G	DNA dC-_dU-editing enzyme APOBEC-3G isoform 2 [Homo sapiens]	−1.82
ARFGAP1	ADP-ribosylation factor GTPase-activating protein 1 isoform c [Homo sapiens]	1.46
ARHGAP24	rho GTPase-activating protein 24 isoform 1 [Homo sapiens]	−1.38
ARHGAP39	rho GTPase-activating protein 39 isoform 1 [Homo sapiens]	−1.86
ARL10	ADP-ribosylation factor-like protein 10 isoform 1 [Homo sapiens]	−1.52
ARRDC4	arrestin domain-containing protein 4 [Homo sapiens]	−2.39
ASB9	ankyrin repeat and SOCS box protein 9 isoform 1 [Homo sapiens]	−3.18
ASF1B	histone chaperone ASF1B [Homo sapiens]	3.19
ASIC1	acid-sensing ion channel 1 isoform b [Homo sapiens]	−2.57
ASPHD1	aspartate beta-hydroxylase domain-containing protein 1 [Homo sapiens]	2.14
ASTN1	astrotactin-1 isoform 3 precursor [Homo sapiens]	4.54
ATF3	cyclic AMP-dependent transcription factor ATF-3 isoform 1 [Homo sapiens]	1.98
ATG10	ubiquitin-like-conjugating enzyme ATG10 [Homo sapiens]	−1.30
ATP10B	probable phospholipid-transporting ATPase VB isoform 1 [Homo sapiens]	−1.62
ATP2B4	plasma membrane calcium-transporting ATPase 4 isoform 4a [Homo sapiens]	−1.98
ATP6	ATP synthase F0 subunit 6 (mitochondrion) [Homo sapiens]	−2.12
ATP6V0A4	V-type proton ATPase 116 kDa subunit a isoform 4 [Homo sapiens]	−1.46
AUTS2	autism susceptibility gene 2 protein isoform 2 [Homo sapiens]	−1.32
B4GALNT1	beta-1,4 N-acetylgalactosaminyltransferase 1 isoform 2 precursor [Homo sapiens]	3.91
BAIAP2L2	brain-specific angiogenesis inhibitor 1-associated protein 2-like protein 2 [Homo sapiens]	−1.56
BANF2	barrier-to-autointegration factor-like protein isoform 1 [Homo sapiens]	3.77

BAX	apoptosis regulator BAX isoform 1 [Homo sapiens]	−3.25
BCAP29	B-cell receptor-associated protein 29 isoform a [Homo sapiens]	−2.95
BCL2	apoptosis regulator Bcl-2 isoform alpha [Homo sapiens]	−1.27
BET1	BET1 homolog isoform 2 [Homo sapiens]	1.34
BFSP1	filensin isoform 2 [Homo sapiens]	−1.43
BHLHE40	class E basic helix-loop-helix protein 40 [Homo sapiens]	−1.51
BHLHE41	class E basic helix-loop-helix protein 41 [Homo sapiens]	−2.22
BNC2	zinc finger protein basonuclin-2 isoform 2 [Homo sapiens]	−1.27
BNIP3	BCL2/adenovirus E1B 19 kDa protein-interacting protein 3 [Homo sapiens]	−1.96
BRSK2	serine/threonine-protein kinase BRSK2 isoform 1 [Homo sapiens]	−1.88
BTLA	B- and T-lymphocyte attenuator isoform 2 [Homo sapiens]	6.01
BTNL8	butyrophilin-like protein 8 isoform 2 precursor [Homo sapiens]	2.93
C10orf90	(E2-independent) E3 ubiquitin-conjugating enzyme FATS isoform 2 [Homo sapiens]	−1.50
C12orf66	KICSTOR complex protein C12orf66 isoform 1 [Homo sapiens]	−1.63
C1orf198	uncharacterized protein C1orf198 isoform 2 [Homo sapiens]	1.27
C1orf21	uncharacterized protein C1orf21 [Homo sapiens]	−1.53
C1orf52	UPF0690 protein C1orf52 [Homo sapiens]	1.32
C1QTNF3	complement C1q tumor necrosis factor-related protein 3 isoform a precursor [Homo sapiens]	−1.71
C1QTNF7	complement C1q tumor necrosis factor-related protein 7 isoform a precursor [Homo sapiens]	1.76
C2orf76	UPF0538 protein C2orf76 isoform 1 [Homo sapiens]	1.54
C3	complement C3 preproprotein [Homo sapiens]	−2.53
C3orf18	uncharacterized protein C3orf18 isoform 1 [Homo sapiens]	−4.50
C3orf52	TPA-induced transmembrane protein isoform 1 [Homo sapiens]	1.49
C3orf85	uncharacterized protein C3orf85 precursor [Homo sapiens]	−2.21
C4orf47	UPF0602 protein C4orf47 isoform 1 [Homo sapiens]	−3.02
C8orf86	uncharacterized protein C8orf86 isoform 2 [Homo sapiens]	4.54
CA10	carbonic anhydrase-related protein 10 precursor [Homo sapiens]	2.87
CAB39L	calcium-binding protein 39-like [Homo sapiens]	1.99
CABLES1	CDK5 and ABL1 enzyme substrate 1 isoform 2 [Homo sapiens]	−1.54
CACNA1D	voltage-dependent L-type calcium channel subunit alpha-1D isoform a [Homo sapiens]	−3.55
CACNA1E	voltage-dependent R-type calcium channel subunit alpha-1E isoform 3 [Homo sapiens]	2.97
CACNA1I	voltage-dependent T-type calcium channel subunit alpha-1I isoform b [Homo sapiens]	2.75
CACNA2D2	voltage-dependent calcium channel subunit alpha-2/delta-2 isoform a precursor [Homo sapiens]	6.07
CACNA2D4	voltage-dependent calcium channel subunit alpha-2/delta-4 [Homo sapiens]	−3.63
CACTIN	cactin [Homo sapiens]	1.69
CADM1	cell adhesion molecule 1 isoform E precursor [Homo sapiens]	−1.37
CAMK4	calcium/calmodulin-dependent protein kinase type IV isoform 1 [Homo sapiens]	−1.35
CAPN3	calpain-3 isoform a [Homo sapiens]	−1.41
CARD14	caspase recruitment domain-containing protein 14 isoform 3 [Homo sapiens]	−2.92
CASP3	caspase-3 isoform a preproprotein [Homo sapiens]	1.39
CASP8	caspase-8 isoform C precursor [Homo sapiens]	1.64
CASP9	caspase-9 isoform alpha precursor [Homo sapiens]	−1.10
CASS4	cas scaffolding protein family member 4 isoform b [Homo sapiens]	2.11
CATSPERE	cation channel sperm-associated protein subunit epsilon isoform 1 precursor [Homo sapiens]	−1.83
CAVIN1	caveolae-associated protein 1 [Homo sapiens]	2.12
CBS	cystathionine beta-synthase isoform 1 [Homo sapiens]	−2.46
CCDC144NL	putative coiled-coil domain-containing protein 144 N-terminal-like [Homo sapiens]	−1.31
CCDC171	coiled-coil domain-containing protein 171 isoform 2 [Homo sapiens]	−1.92
CCDC191	coiled-coil domain-containing protein 191 isoform 2 [Homo sapiens]	−1.65
CCDC196	putative coiled-coil domain-containing protein 196 [Homo sapiens]	−2.22
CCDC57	coiled-coil domain-containing protein 57 isoform 2 [Homo sapiens]	−1.41
CCDC7	coiled-coil domain-containing protein 7 isoform a [Homo sapiens]	−1.52
CCN1	CCN family member 1 precursor [Homo sapiens]	2.40
CCN3	CCN family member 3 precursor [Homo sapiens]	−2.76
CDCP1	CUB domain-containing protein 1 isoform 1 precursor [Homo sapiens]	−2.82
CDH17	cadherin-17 precursor [Homo sapiens]	2.69
CDHR3	cadherin-related family member 3 isoform 2 [Homo sapiens]	−1.42
CDK15	cyclin-dependent kinase 15 isoform 1 [Homo sapiens]	4.39
CDK7	cyclin-dependent kinase 7 isoform 2 [Homo sapiens]	−2.01
CDKN1A	cyclin-dependent kinase inhibitor 1 isoform 1 [Homo sapiens]	1.82
CEACAM8	carcinoembryonic antigen-related cell adhesion molecule 8 precursor [Homo sapiens]	3.54
CELSR2	cadherin EGF LAG seven-pass G-type receptor 2 precursor [Homo sapiens]	−2.07

CEP170	centrosomal protein of 170 kDa isoform beta [Homo sapiens]	−2.66
CFAP44	cilia- and flagella-associated protein 44 isoform 1 [Homo sapiens]	−2.11
CFAP45	cilia- and flagella-associated protein 45 [Homo sapiens]	3.00
CHL1	neural cell adhesion molecule L1-like protein isoform 2 precursor [Homo sapiens]	−1.88
CHRM3	muscarinic acetylcholine receptor M3 [Homo sapiens]	−1.28
CHST11	carbohydrate sulfotransferase 11 isoform 2 [Homo sapiens]	−1.69
CHST9	carbohydrate sulfotransferase 9 isoform 2 [Homo sapiens]	3.57
CHSY3	chondroitin sulfate synthase 3 [Homo sapiens]	−1.64
CKS2	cyclin-dependent kinases regulatory subunit 2 [Homo sapiens]	1.21
CLCF1	cardiotrophin-like cytokine factor 1 isoform 2 precursor [Homo sapiens]	2.71
CLDN14	claudin-14 [Homo sapiens]	−1.81
CLMN	calmin [Homo sapiens]	−1.64
CLU	clusterin preproprotein [Homo sapiens]	4.40
CNIH3	protein cornichon homolog 3 isoform 2 [Homo sapiens]	−1.71
CNTN1	contactin-1 isoform 3 precursor [Homo sapiens]	−2.14
CNTN3	contactin-3 precursor [Homo sapiens]	2.97
CNTNAP3	contactin-associated protein-like 3 precursor [Homo sapiens]	-1.33
CNTNAP3C	contactin-associated protein-like 3 isoform X2 [Homo sapiens]	−2.20
COL16A1	collagen alpha-1(XVI) chain precursor [Homo sapiens]	−1.69
COL27A1	collagen alpha-1(XXVII) chain preproprotein [Homo sapiens]	−2.44
COL5A3	collagen alpha-3(V) chain preproprotein [Homo sapiens]	−2.83
COLEC12	collectin-12 [Homo sapiens]	3.91
COX2	cytochrome c oxidase subunit II (mitochondrion) [Homo sapiens]	−1.34
COX3	cytochrome c oxidase subunit III (mitochondrion) [Homo sapiens]	−1.78
CPA6	carboxypeptidase A6 preproprotein [Homo sapiens]	4.36
CPHXL	cytoplasmic polyadenylated homeobox-like [Homo sapiens]	4.62
CPQ	carboxypeptidase Q preproprotein [Homo sapiens]	−1.62
CSGALNACT1	chondroitin sulfate N-acetylgalactosaminyltransferase 1 [Homo sapiens]	−2.06
CSPG4	chondroitin sulfate proteoglycan 4 precursor [Homo sapiens]	−2.27
CSRNP1	cysteine/serine-rich nuclear protein 1 isoform a [Homo sapiens]	1.53
CTDSP1	carboxy-terminal domain RNA polymerase II polypeptide A small phosphatase 1 isoform 3 [Homo sapiens]	−2.31
CTDSPL	CTD small phosphatase-like protein isoform 1 [Homo sapiens]	−1.29
CTLA4	cytotoxic T-lymphocyte protein 4 isoform CTLA-4delTM [Homo sapiens]	−3.29
CTTN	src substrate cortactin isoform c [Homo sapiens]	−1.99
CUTA	protein CutA isoform 1 [Homo sapiens]	−3.18
CXCL1	growth-regulated alpha protein precursor [Homo sapiens]	−4.45
CXCL8	interleukin-8 isoform 1 precursor [Homo sapiens]	−2.66
CYP26B1	cytochrome P450 26B1 isoform 2 [Homo sapiens]	−3.44
CYP4V2	cytochrome P450 4V2 [Homo sapiens]	−2.75
CYP4Z1	cytochrome P450 4Z1 [Homo sapiens]	−4.41
CYP7B1	25-hydroxycholesterol 7-alpha-hydroxylase isoform 2 [Homo sapiens]	−1.24
CYTB	cytochrome b (mitochondrion) [Homo sapiens]	−2.14
DAPK3	death-associated protein kinase 3 [Homo sapiens]	1.78
DCT	L-dopachrome tautomerase isoform 2 precursor [Homo sapiens]	−2.90
DGKG	diacylglycerol kinase gamma isoform 2 [Homo sapiens]	−1.66
DGKI	diacylglycerol kinase iota isoform 2 [Homo sapiens]	−2.38
DHFR2	dihydrofolate reductase 2, mitochondrial [Homo sapiens]	−2.24
DHRS2	dehydrogenase/reductase SDR family member 2, mitochondrial isoform 3 [Homo sapiens]	−3.42
DHRS4	dehydrogenase/reductase SDR family member 4 isoform 2 [Homo sapiens]	−4.55
DHX38	pre-mRNA-splicing factor ATP-dependent RNA helicase PRP16 [Homo sapiens]	1.29
DISC1	disrupted in schizophrenia 1 protein isoform Lv [Homo sapiens]	−1.42
DMTN	dematin isoform 1 [Homo sapiens]	2.67
DNAAF4	dynein assembly factor 4, axonemal isoform b [Homo sapiens]	−1.62
DNAH14	dynein heavy chain 14, axonemal isoform 2 [Homo sapiens]	−1.57
DNAH17	dynein heavy chain 17, axonemal [Homo sapiens]	2.08
DNAH3	dynein heavy chain 3, axonemal isoform 2 [Homo sapiens]	2.27
DNAJB1	dnaJ homolog subfamily B member 1 isoform 2 [Homo sapiens]	1.39
DNER	delta and Notch-like epidermal growth factor-related receptor precursor [Homo sapiens]	−1.37
DOK3	docking protein 3 isoform 2 [Homo sapiens]	−2.11
DPP10	inactive dipeptidyl peptidase 10 isoform short [Homo sapiens]	3.66
DPP6	dipeptidyl aminopeptidase-like protein 6 isoform 3 [Homo sapiens]	−1.70
DPYSL2	dihydropyrimidinase-related protein 2 isoform 1 [Homo sapiens]	−1.23

DRAXIN	draxin precursor [Homo sapiens]	−3.48
DSCAM	Down syndrome cell adhesion molecule isoform 2 precursor [Homo sapiens]	3.58
DSE	dermatan-sulfate epimerase isoform a precursor [Homo sapiens]	−1.30
DTWD2	DTW domain-containing protein 2 isoform b [Homo sapiens]	−1.32
DTX4	E3 ubiquitin-protein ligase DTX4 isoform 2 [Homo sapiens]	−3.06
DUSP2	dual specificity protein phosphatase 2 [Homo sapiens]	2.74
E2F5	transcription factor E2F5 isoform 2 [Homo sapiens]	−1.91
EBF3	transcription factor COE3 [Homo sapiens]	−1.55
EDA	ectodysplasin-A isoform 2 [Homo sapiens]	−2.17
EEF2K	eukaryotic elongation factor 2 kinase [Homo sapiens]	−3.27
EFR3B	protein EFR3 homolog B isoform 2 [Homo sapiens]	−2.09
EGLN1	egl nine homolog 1 [Homo sapiens]	−1.67
EGR1	early growth response protein 1 [Homo sapiens]	2.44
EHF	ETS homologous factor isoform 3 [Homo sapiens]	−1.70
EIF3C	eukaryotic translation initiation factor 3 subunit C isoform a [Homo sapiens]	−1.61
EIF3CL	eukaryotic translation initiation factor 3 subunit C-like protein [Homo sapiens]	−1.81
EMILIN2	EMILIN-2 precursor [Homo sapiens]	−1.75
ENDOD1	endonuclease domain-containing 1 protein precursor [Homo sapiens]	−1.85
ENO2	gamma-enolase [Homo sapiens]	−2.36
ENOX1	ecto-NOX disulfide-thiol exchanger 1 isoform b [Homo sapiens]	−1.52
ENPP3	ectonucleotide pyrophosphatase/phosphodiesterase family member 3 [Homo sapiens]	−2.09
ENPP5	ectonucleotide pyrophosphatase/phosphodiesterase family member 5 isoform 1 precursor [Homo sapiens]	−1.73
EPB41L1	band 4.1-like protein 1 isoform c [Homo sapiens]	−1.60
EPHA3	ephrin type-A receptor 3 isoform a precursor [Homo sapiens]	−2.57
EPHA6	ephrin type-A receptor 6 isoform a [Homo sapiens]	2.94
EPHB3	ephrin type-B receptor 3 precursor [Homo sapiens]	-2.66
ERBB3	receptor tyrosine-protein kinase erbB-3 isoform s precursor [Homo sapiens]	−2.86
ERC2	ERC protein 2 [Homo sapiens]	3.26
ERG	transcriptional regulator ERG isoform 3 [Homo sapiens]	3.62
ERMN	ermin isoform a [Homo sapiens]	−3.94
ERO1A	ERO1-like protein alpha precursor [Homo sapiens]	−1.51
ERVH48-1	suppressyn precursor [Homo sapiens]	3.91
ESPN	espin isoform 2 [Homo sapiens]	−4.66
ESYT3	extended synaptotagmin-3 isoform a [Homo sapiens]	−1.70
ETV6	transcription factor ETV6 [Homo sapiens]	−1.43
ETV7	transcription factor ETV7 isoform 2 [Homo sapiens]	2.29
EXTL1	exostosin-like 1 [Homo sapiens]	−1.63
EYS	protein eyes shut homolog isoform 1 precursor [Homo sapiens]	−1.71
FAAH2	fatty-acid amide hydrolase 2 isoform 2 [Homo sapiens]	−1.62
FAM110B	protein FAM110B [Homo sapiens]	−1.77
FAM117B	protein FAM117B [Homo sapiens]	−2.42
FAM205A	protein FAM205A [Homo sapiens]	4.36
FAM222A	protein FAM222A [Homo sapiens]	−2.14
FAM50A	protein FAM50A [Homo sapiens]	1.57
FAM71F2	protein FAM71F2 isoform a [Homo sapiens]	−2.39
FAM78B	protein FAM78B isoform 1 [Homo sapiens]	−3.54
FAXDC2	fatty acid hydroxylase domain-containing protein 2 [Homo sapiens]	−1.74
FBXL17	F-box/LRR-repeat protein 17 [Homo sapiens]	−1.34
FBXO3	F-box only protein 3 isoform 1 [Homo sapiens]	1.32
FBXO41	F-box only protein 41 [Homo sapiens]	−1.65
FCGR2A	low affinity immunoglobulin gamma Fc region receptor II-a isoform 1 precursor [Homo sapiens]	−2.44
FHDC1	FH2 domain-containing protein 1 [Homo sapiens]	−1.78
FKBP9	peptidyl-prolyl cis-trans isomerase FKBP9 isoform 2 precursor [Homo sapiens]	−1.47
FLG	filaggrin [Homo sapiens]	−1.51
FLII	protein flightless-1 homolog isoform 2 [Homo sapiens]	−1.98
FLNC	filamin-C isoform b [Homo sapiens]	3.04
FOPNL	lisH domain-containing protein FOPNL isoform 2 [Homo sapiens]	−5.25
FOXP1	forkhead box protein P1 isoform b [Homo sapiens]	−1.31
FREM2	FRAS1-related extracellular matrix protein 2 precursor [Homo sapiens]	−1.80
FRG1	protein FRG1 [Homo sapiens]	4.51
FRK	tyrosine-protein kinase FRK [Homo sapiens]	−1.22

FRMD3	FERM domain-containing protein 3 isoform 2 [Homo sapiens]	−2.00
FSIP2	fibrous sheath-interacting protein 2 [Homo sapiens]	−1.57
FSTL4	follistatin-related protein 4 precursor [Homo sapiens]	2.70
FTCDNL1	formiminotransferase N-terminal subdomain-containing protein isoform 1 precursor [Homo sapiens]	−1.82
FTL	ferritin light chain [Homo sapiens]	2.09
FUT11	alpha-(1,3)-fucosyltransferase 11 isoform 2 precursor [Homo sapiens]	−2.01
FUT3	galactoside 3(4)-L-fucosyltransferase [Homo sapiens]	−3.22
FYB1	FYN-binding protein 1 isoform 3 [Homo sapiens]	−1.94
GAB2	GRB2-associated-binding protein 2 isoform b [Homo sapiens]	−1.61
GABARAPL1	gamma-aminobutyric acid receptor-associated protein-like 1 isoform 1 [Homo sapiens]	1.32
GABRB2	gamma-aminobutyric acid receptor subunit beta-2 isoform 2 precursor [Homo sapiens]	4.54
GADD45B	growth arrest and DNA damage-inducible protein GADD45 beta [Homo sapiens]	1.89
GALNT3	polypeptide N-acetylgalactosaminyltransferase 3 [Homo sapiens]	−2.12
GAREM1	GRB2-associated and regulator of MAPK protein 1 isoform 1 [Homo sapiens]	−1.31
GAS1	growth arrest-specific protein 1 precursor [Homo sapiens]	−3.04
GAS7	growth arrest-specific protein 7 isoform d [Homo sapiens]	−1.51
GCM2	chorion-specific transcription factor GCMb [Homo sapiens]	5.21
GDAP1	ganglioside-induced differentiation-associated protein 1 isoform b [Homo sapiens]	−1.55
GEM	GTP-binding protein GEM [Homo sapiens]	1.93
GGT1	glutathione hydrolase 1 proenzyme precursor [Homo sapiens]	3.12
GLRA1	glycine receptor subunit alpha-1 isoform 2 precursor [Homo sapiens]	−1.69
GMPR2	GMP reductase 2 isoform 2 [Homo sapiens]	2.93
GNAO1	guanine nucleotide-binding protein G(o) subunit alpha isoform a [Homo sapiens]	−1.89
GPC6	glypican-6 precursor [Homo sapiens]	−2.07
GPM6B	neuronal membrane glycoprotein M6-b isoform 4 [Homo sapiens]	−1.27
GPR149	probable G-protein coupled receptor 149 [Homo sapiens]	5.26
GPR15	G-protein coupled receptor 15 [Homo sapiens]	2.38
GPR153	probable G-protein coupled receptor 153 [Homo sapiens]	−1.83
GPR155	integral membrane protein GPR155 isoform 1 [Homo sapiens]	−1.53
GPR158	probable G-protein coupled receptor 158 precursor [Homo sapiens]	−1.22
GPR161	G-protein coupled receptor 161 isoform 1 [Homo sapiens]	−1.48
GPRC5B	G-protein coupled receptor family C group 5 member B isoform 2 [Homo sapiens]	−1.48
GRAMD1B	protein Aster-B isoform 1 [Homo sapiens]	−1.85
GRASP	general receptor for phosphoinositides 1-associated scaffold protein isoform 2 [Homo sapiens]	−1.71
GREB1L	GREB1-like protein [Homo sapiens]	−1.53
GRK5	G protein-coupled receptor kinase 5 [Homo sapiens]	−1.47
GRXCR2	glutaredoxin domain-containing cysteine-rich protein 2 [Homo sapiens]	4.48
GTDC1	glycosyltransferase-like domain-containing protein 1 isoform a [Homo sapiens]	−1.36
GTF2IRD2B	general transcription factor II-I repeat domain-containing protein 2B isoform 1 [Homo sapiens]	−2.20
GUCA1C	guanylyl cyclase-activating protein 3 isoform 2 [Homo sapiens]	−3.01
GYG2	glycogenin-2 isoform a [Homo sapiens]	−1.71
H3F3C	histone H3.3C [Homo sapiens]	5.80
HACD4	very-long-chain (3R)-3-hydroxyacyl-CoA dehydratase 4 isoform 1 [Homo sapiens]	−2.01
HAPLN3	hyaluronan and proteoglycan link protein 3 isoform 1 [Homo sapiens]	2.08
HAS2	hyaluronan synthase 2 [Homo sapiens]	−3.39
HGD	homogentisate 1,2-dioxygenase [Homo sapiens]	4.62
HGSNAT	heparan-alpha-glucosaminide N-acetyltransferase isoform 2 precursor [Homo sapiens]	−1.54
HIST1H1A	histone H1.1 [Homo sapiens]	−2.28
HIST1H1B	histone H1.5 [Homo sapiens]	−1.58
HIST1H2AJ	histone cluster 1, H2aj [Homo sapiens]	−1.89
HIST1H2BF	histone H2B type 1-C/E/F/G/I [Homo sapiens]	−1.51
HIST1H2BM	histone H2B type 1-M [Homo sapiens]	−2.30
HIST1H3A	histone H3.1 [Homo sapiens]	−1.73
HIST1H3D	histone H3.1 [Homo sapiens]	−1.90
HIST1H3H	histone H3.1 [Homo sapiens]	−1.67
HIST2H2AA3	histone H2A type 2-A [Homo sapiens]	−2.37
HIST2H2AC	histone H2A type 2-C [Homo sapiens]	−1.28
HIST2H3C	histone H3.2 [Homo sapiens]	−2.35
HIST2H4A	histone H4 [Homo sapiens]	−3.33
HIVEP3	transcription factor HIVEP3 isoform b [Homo sapiens]	−1.40
HLCS	biotin--protein ligase isoform 1 [Homo sapiens]	−1.44

HMCN1	hemicentin-1 precursor [Homo sapiens]	−2.07
HMOX1	heme oxygenase 1 [Homo sapiens]	1.87
HOXA13	homeobox protein Hox-A13 [Homo sapiens]	−4.48
HOXB3	homeobox protein Hox-B3 isoform 2 [Homo sapiens]	−1.87
HSPB8	heat shock protein beta-8 [Homo sapiens]	2.15
HSPE1-MOB4	HSPE1-MOB4 protein [Homo sapiens]	1.99
HSPH1	heat shock protein 105 kDa isoform 2 [Homo sapiens]	1.78
HTRA1	serine protease HTRA1 precursor [Homo sapiens]	−1.83
IBTK	inhibitor of Bruton tyrosine kinase isoform 2 [Homo sapiens]	−3.36
IDH1	isocitrate dehydrogenase [NADP] cytoplasmic [Homo sapiens]	−1.29
IFIT1	interferon-induced protein with tetratricopeptide repeats 1 isoform 2 [Homo sapiens]	−2.49
IGF1	insulin-like growth factor I isoform 4 preproprotein [Homo sapiens]	−1.32
IGF1R	insulin-like growth factor 1 receptor isoform 1 precursor [Homo sapiens]	−1.33
IGFBP3	insulin-like growth factor-binding protein 3 isoform b precursor [Homo sapiens]	−4.99
IGFN1	immunoglobulin-like and fibronectin type III domain-containing protein 1 isoform 1 [Homo sapiens]	1.42
IGSF11	immunoglobulin superfamily member 11 isoform b precursor [Homo sapiens]	−2.53
IL11	interleukin-11 isoform 1 precursor [Homo sapiens]	1.47
IL18R1	interleukin-18 receptor 1 isoform 2 [Homo sapiens]	−1.72
IL1RAP	interleukin-1 receptor accessory protein isoform 1 precursor [Homo sapiens]	−1.72
IL6R	interleukin-6 receptor subunit alpha isoform 1 precursor [Homo sapiens]	2.65
IMPACT	protein IMPACT [Homo sapiens]	−1.34
INSIG1	insulin-induced gene 1 protein isoform 4 [Homo sapiens]	1.40
INTS3	integrator complex subunit 3 [Homo sapiens]	−2.36
IPO4	importin-4 [Homo sapiens]	−2.47
IQCM	IQ domain-containing protein M isoform 1 [Homo sapiens]	−1.97
IRF4	interferon regulatory factor 4 isoform 2 [Homo sapiens]	−2.16
ITGA2	integrin alpha-2 precursor [Homo sapiens]	−1.34
ITGB2	integrin beta-2 isoform 1 precursor [Homo sapiens]	4.00
ITGB3	integrin beta-3 precursor [Homo sapiens]	−1.46
ITGB8	integrin beta-8 precursor [Homo sapiens]	−2.22
ITIH6	inter-alpha-trypsin inhibitor heavy chain H6 precursor [Homo sapiens]	−2.65
JAG1	protein jagged-1 precursor [Homo sapiens]	−1.37
JUNB	transcription factor jun-B [Homo sapiens]	1.68
KAT2B	histone acetyltransferase KAT2B [Homo sapiens]	−1.23
KBTBD8	kelch repeat and BTB domain-containing protein 8 [Homo sapiens]	1.46
KCNB2	potassium voltage-gated channel subfamily B member 2 [Homo sapiens]	3.70
KCNH5	potassium voltage-gated channel subfamily H member 5 isoform 1 [Homo sapiens]	−1.35
KCNH7	potassium voltage-gated channel subfamily H member 7 isoform 1 [Homo sapiens]	2.82
KCNH8	potassium voltage-gated channel subfamily H member 8 [Homo sapiens]	−1.31
KCNIP4	Kv channel-interacting protein 4 isoform 5 [Homo sapiens]	−1.52
KCNK13	potassium channel subfamily K member 13 [Homo sapiens]	−1.75
KCNK2	potassium channel subfamily K member 2 isoform a [Homo sapiens]	−2.08
KCNN4	intermediate conductance calcium-activated potassium channel protein 4 [Homo sapiens]	−1.72
KCNQ3	potassium voltage-gated channel subfamily KQT member 3 isoform 2 precursor [Homo sapiens]	−2.93
KCNS3	potassium voltage-gated channel subfamily S member 3 [Homo sapiens]	−1.55
KCTD12	BTB/POZ domain-containing protein KCTD12 [Homo sapiens]	−3.99
KCTD5	BTB/POZ domain-containing protein KCTD5 [Homo sapiens]	1.28
KDR	vascular endothelial growth factor receptor 2 precursor [Homo sapiens]	−2.70
KIAA0825	uncharacterized protein KIAA0825 isoform 1 [Homo sapiens]	−1.60
KIAA1549L	UPF0606 protein KIAA1549L [Homo sapiens]	−1.98
KIF5C	kinesin heavy chain isoform 5C [Homo sapiens]	−6.22
KLF7	Krueppel-like factor 7 isoform 2 [Homo sapiens]	−1.71
KLHDC8A	kelch domain-containing protein 8A [Homo sapiens]	4.56
KLHL35	kelch-like protein 35 [Homo sapiens]	2.79
KLRD1	natural killer cells antigen CD94 isoform 1 [Homo sapiens]	3.80
KLRG2	killer cell lectin-like receptor subfamily G member 2 [Homo sapiens]	−2.60
KSR2	kinase suppressor of Ras 2 [Homo sapiens]	−2.24
KYAT3	kynurenine--oxoglutarate transaminase 3 isoform 1 [Homo sapiens]	1.36
L1CAM	neural cell adhesion molecule L1 isoform 1 precursor [Homo sapiens]	−1.86
L2HGDH	L-2-hydroxyglutarate dehydrogenase, mitochondrial precursor [Homo sapiens]	1.27
L3MBTL4	lethal(3)malignant brain tumor-like protein 4 isoform 2 [Homo sapiens]	−1.30

LAMA4	laminin subunit alpha-4 isoform 1 precursor [Homo sapiens]	−1.36
LAPTM5	lysosomal-associated transmembrane protein 5 [Homo sapiens]	−2.04
LCN2	neutrophil gelatinase-associated lipocalin precursor [Homo sapiens]	−2.12
LDB3	LIM domain-binding protein 3 isoform 2 [Homo sapiens]	−1.90
LDLRAD4	low-density lipoprotein receptor class A domain-containing protein 4 isoform gamma 1 [Homo sapiens]	−1.63
LGI3	leucine-rich repeat LGI family member 3 precursor [Homo sapiens]	4.62
LHFPL3	LHFPL tetraspan subfamily member 3 protein [Homo sapiens]	−2.95
LINGO2	leucine-rich repeat and immunoglobulin-like domain-containing nogo receptor-interacting protein 2 precursor [Homo sapiens]	−1.45
LINGO3	leucine-rich repeat and immunoglobulin-like domain-containing nogo receptor-interacting protein 3 precursor [Homo sapiens]	3.74
LOC100130370	uncharacterized protein LOC100130370 isoform 1 [Homo sapiens]	−2.69
LOC100506422	putative inactive deoxyuridine 5'-triphosphate nucleotidohydrolase-like protein FLJ16323 [Homo sapiens]	−1.46
LOC101059949	putative ankyrin repeat domain-containing protein 20A12 pseudogene [Homo sapiens]	−3.46
LOC101927345	putative ankyrin repeat domain-containing protein 20A12 pseudogene [Homo sapiens]	−1.83
LOC105375817	POTE ankyrin domain family member A-like [Homo sapiens]	−1.92
LOC112268284	basic proline-rich protein-like [Homo sapiens]	−3.19
LOXL2	lysyl oxidase homolog 2 precursor [Homo sapiens]	−2.25
LPAR3	lysophosphatidic acid receptor 3 [Homo sapiens]	−1.76
LRGUK	leucine-rich repeat and guanylate kinase domain-containing protein isoform 1 [Homo sapiens]	−1.97
LRIG3	leucine-rich repeats and immunoglobulin-like domains protein 3 isoform 1 precursor [Homo sapiens]	−1.62
LRMDA	leucine-rich melanocyte differentiation-associated protein isoform 1 [Homo sapiens]	−1.65
LRP1	prolow-density lipoprotein receptor-related protein 1 preproprotein [Homo sapiens]	−2.55
LRP1B	low-density lipoprotein receptor-related protein 1B precursor [Homo sapiens]	2.25
LRRC6	protein tilB homolog isoform b [Homo sapiens]	−1.62
LRRC8D	volume-regulated anion channel subunit LRRC8D [Homo sapiens]	−1.41
LSAMP	limbic system-associated membrane protein isoform 2 precursor [Homo sapiens]	−3.13
LSMEM1	leucine-rich single-pass membrane protein 1 [Homo sapiens]	1.64
LTF	lactotransferrin isoform 2 [Homo sapiens]	2.15
LZTS1	leucine zipper putative tumor suppressor 1 [Homo sapiens]	−2.35
MAFK	transcription factor MafK [Homo sapiens]	1.53
MAGI2	membrane-associated guanylate kinase, WW and PDZ domain-containing protein 2 isoform 2 [Homo sapiens]	−1.48
MAN1A1	mannosyl-oligosaccharide 1,2-alpha-mannosidase IA [Homo sapiens]	2.03
MAP2K6	dual specificity mitogen-activated protein kinase kinase 6 isoform 2 [Homo sapiens]	−3.88
MAP3K6	mitogen-activated protein kinase kinase kinase 6 isoform 2 [Homo sapiens]	1.54
MAP7D2	MAP7 domain-containing protein 2 isoform 1 [Homo sapiens]	4.47
MARCKSL1	MARCKS-related protein [Homo sapiens]	−1.92
MBLAC2	metallo-beta-lactamase domain-containing protein 2 [Homo sapiens]	−1.65
MCC	colorectal mutant cancer protein isoform 1 [Homo sapiens]	−1.23
MCTP2	multiple C2 and transmembrane domain-containing protein 2 isoform 2 [Homo sapiens]	−1.57
MDGA2	MAM domain-containing glycosylphosphatidylinositol anchor protein 2 isoform 1 precursor [Homo sapiens]	−1.47
ME3	NADP-dependent malic enzyme, mitochondrial [Homo sapiens]	1.73
MFSD2B	major facilitator superfamily domain-containing protein 2B [Homo sapiens]	1.85
MGMT	methylated-DNA--protein-cysteine methyltransferase [Homo sapiens]	−1.32
MLLT3	protein AF-9 isoform b [Homo sapiens]	−1.52
MMP17	matrix metalloproteinase-17 preproprotein [Homo sapiens]	−1.54
MMP24-AS1-EDEM2	ER degradation-enhancing alpha-mannosidase-like protein 2 [Homo sapiens]	3.41
MORC1	MORC family CW-type zinc finger protein 1 [Homo sapiens]	−2.49
MORN1	MORN repeat-containing protein 1 isoform 2 [Homo sapiens]	−1.60
MREG	melanoregulin [Homo sapiens]	−1.42
MRGPRX3	mas-related G-protein coupled receptor member X3 [Homo sapiens]	−2.92
MRPL41	39S ribosomal protein L41, mitochondrial [Homo sapiens]	1.65
MRTFB	myocardin-related transcription factor B isoform 1 [Homo sapiens]	−1.30
MTCH1	mitochondrial carrier homolog 1 isoform PSAP-LL [Homo sapiens]	1.38
MTCH2	mitochondrial carrier homolog 2 isoform 1x [Homo sapiens]	3.02
MXI1	max-interacting protein 1 isoform c [Homo sapiens]	−1.56
MYH15	myosin-15 precursor [Homo sapiens]	−1.65

MYL6	myosin light polypeptide 6 isoform 1 [Homo sapiens]	1.83
MYO3A	myosin-IIIa isoform 2 [Homo sapiens]	4.91
MYOM1	myomesin-1 isoform a [Homo sapiens]	1.78
NAP1L4	nucleosome assembly protein 1-like 4 isoform 1 [Homo sapiens]	−3.72
NAV2	neuron navigator 2 isoform 3 [Homo sapiens]	−1.27
NAV3	neuron navigator 3 isoform 1 [Homo sapiens]	1.31
NCF2	neutrophil cytosol factor 2 isoform 1 [Homo sapiens]	−2.33
NCK2	cytoplasmic protein NCK2 isoform A [Homo sapiens]	−1.33
NCKAP5	nck-associated protein 5 isoform 1 [Homo sapiens]	−1.46
NCOA5	nuclear receptor coactivator 5 isoform 2 [Homo sapiens]	−1.89
ND2	NADH dehydrogenase subunit 2 (mitochondrion) [Homo sapiens]	−1.59
ND3	NADH dehydrogenase subunit 3 (mitochondrion) [Homo sapiens]	−1.92
ND4L	NADH dehydrogenase subunit 4L (mitochondrion) [Homo sapiens]	−1.76
ND5	NADH dehydrogenase subunit 5 (mitochondrion) [Homo sapiens]	−1.35
NDRG1	protein NDRG1 isoform 1 [Homo sapiens]	−1.97
NEDD9	enhancer of filamentation 1 isoform 3 [Homo sapiens]	−1.60
NEMF	nuclear export mediator factor NEMF isoform 2 [Homo sapiens]	1.47
NFASC	neurofascin isoform 1 precursor [Homo sapiens]	3.97
NIPSNAP2	protein NipSnap homolog 2 isoform 2 [Homo sapiens]	−1.39
NKAIN4	sodium/potassium-transporting ATPase subunit beta-1-interacting protein 4 isoform 2 [Homo sapiens]	−2.94
NLGN1	neuroligin-1 isoform 1 precursor [Homo sapiens]	−1.86
NLRP1	NACHT, LRR and PYD domains-containing protein 1 isoform 5 [Homo sapiens]	1.34
NPAS2	neuronal PAS domain-containing protein 2 [Homo sapiens]	−1.64
NPPC	C-type natriuretic peptide preproprotein [Homo sapiens]	−2.71
NQO1	NAD(P)H dehydrogenase [quinone] 1 isoform a [Homo sapiens]	1.52
NR3C2	mineralocorticoid receptor isoform 1 [Homo sapiens]	−1.61
NR4A1	nuclear receptor subfamily 4 group A member 1 isoform 2 [Homo sapiens]	1.36
NRG2	pro-neuregulin-2, membrane-bound isoform isoform 7 [Homo sapiens]	−2.30
NRN1	neuritin isoform 1 precursor [Homo sapiens]	−2.01
NRP1	neuropilin-1 isoform b precursor [Homo sapiens]	−2.01
NRP2	neuropilin-2 isoform 2 precursor [Homo sapiens]	−1.62
NTM	neurotrimin isoform 2 precursor [Homo sapiens]	−1.65
NUP160	nuclear pore complex protein Nup160 isoform 2 [Homo sapiens]	−1.95
OCA2	P protein isoform 1 [Homo sapiens]	−2.67
ODF2L	protein BCAP isoform b [Homo sapiens]	−1.51
OMA1	metalloendopeptidase OMA1, mitochondrial precursor [Homo sapiens]	−1.49
ONECUT1	hepatocyte nuclear factor 6 [Homo sapiens]	−2.55
ONECUT2	one cut domain family member 2 [Homo sapiens]	−1.53
OPN5	opsin-5 [Homo sapiens]	−1.95
OR2L13	olfactory receptor 2L13 [Homo sapiens]	−1.96
OR9Q1	olfactory receptor 9Q1 [Homo sapiens]	−3.08
OSBP2	oxysterol-binding protein 2 isoform c [Homo sapiens]	−1.40
OTOGL	otogelin-like protein isoform 2 precursor [Homo sapiens]	2.13
OVGP1	oviduct-specific glycoprotein precursor [Homo sapiens]	2.18
PAGE5	P antigen family member 5 isoform 2 [Homo sapiens]	1.32
PANX1	pannexin-1 [Homo sapiens]	−1.51
PAPPA	pappalysin-1 preproprotein [Homo sapiens]	−1.91
PAQR8	membrane progestin receptor beta [Homo sapiens]	−1.52
PARN	poly(A)-specific ribonuclease PARN isoform 2 [Homo sapiens]	−2.11
PCDH15	protocadherin-15 isoform CD1-1 precursor [Homo sapiens]	−1.61
PDE4B	cAMP-specific 3',5'-cyclic phosphodiesterase 4B isoform 2 [Homo sapiens]	−2.19
PDGFC	platelet-derived growth factor C precursor [Homo sapiens]	−1.29
PDGFD	platelet-derived growth factor D isoform 1 precursor [Homo sapiens]	−1.94
PDK1	[Pyruvate dehydrogenase (acetyl-transferring)] kinase isozyme 1, mitochondrial isoform 1 precursor [Homo sapiens]	−2.05
PDZRN4	PDZ domain-containing RING finger protein 4 isoform 1 [Homo sapiens]	4.04
PEBP4	phosphatidylethanolamine-binding protein 4 precursor [Homo sapiens]	−1.78
PER2	period circadian protein homolog 2 [Homo sapiens]	−5.13
PER3	period circadian protein homolog 3 isoform 2 [Homo sapiens]	−1.84
PFKFB4	6-phosphofructo-2-kinase/fructose-2,6-bisphosphatase 4 isoform a [Homo sapiens]	−2.22
PGM1	phosphoglucomutase-1 isoform 2 [Homo sapiens]	−1.34
PHF10	PHD finger protein 10 isoform a [Homo sapiens]	−1.38

PHF14	PHD finger protein 14 isoform 1 [Homo sapiens]	−1.57
PHLDA1	pleckstrin homology-like domain family A member 1 [Homo sapiens]	−1.25
PID1	PTB-containing, cubilin and LRP1-interacting protein isoform 2 [Homo sapiens]	−1.71
PIEZO2	piezo-type mechanosensitive ion channel component 2 [Homo sapiens]	−1.63
PIK3R1	phosphatidylinositol 3-kinase regulatory subunit alpha isoform 4 [Homo sapiens]	−1.45
PITPNC1	cytoplasmic phosphatidylinositol transfer protein 1 isoform a [Homo sapiens]	−2.03
PKNOX2	homeobox protein PKNOX2 [Homo sapiens]	−1.94
PLA2G6	85/88 kDa calcium-independent phospholipase A2 isoform b [Homo sapiens]	−1.28
PLB1	phospholipase B1, membrane-associated isoform 2 precursor [Homo sapiens]	−3.13
PLCB4	1-phosphatidylinositol 4,5-bisphosphate phosphodiesterase beta-4 isoform a [Homo sapiens]	−1.51
PLEKHA2	pleckstrin homology domain-containing family A member 2 [Homo sapiens]	−1.52
PLEKHG1	pleckstrin homology domain-containing family G member 1 isoform c [Homo sapiens]	−2.25
PLPP3	phospholipid phosphatase 3 [Homo sapiens]	−2.12
PLPP4	phospholipid phosphatase 4 isoform a [Homo sapiens]	−1.57
PNPLA7	patatin-like phospholipase domain-containing protein 7 isoform a [Homo sapiens]	−2.31
POGLUT2	protein O-glucosyltransferase 2 isoform 2 [Homo sapiens]	−2.33
POLR2F	DNA-directed RNA polymerases I, II, and III subunit RPABC2 isoform 2 [Homo sapiens]	−1.57
POLR3E	DNA-directed RNA polymerase III subunit RPC5 isoform 2 [Homo sapiens]	−2.22
POSTN	periostin isoform 2 precursor [Homo sapiens]	5.42
POTEM	putative POTE ankyrin domain family member M [Homo sapiens]	−2.62
POU3F2	POU domain, class 3, transcription factor 2 [Homo sapiens]	−1.43
PPARGC1A	peroxisome proliferator-activated receptor gamma coactivator 1-alpha isoform 1 [Homo sapiens]	−1.75
PPARGC1B	peroxisome proliferator-activated receptor gamma coactivator 1-beta isoform 2 [Homo sapiens]	−1.90
PPFIA1	liprin-alpha-1 isoform b [Homo sapiens]	−3.46
PPFIA2	liprin-alpha-2 isoform b [Homo sapiens]	−1.58
PPFIA4	liprin-alpha-4 isoform 1 [Homo sapiens]	−3.13
PPL	periplakin [Homo sapiens]	−1.75
PPM1H	protein phosphatase 1H [Homo sapiens]	−1.81
PPP1R15A	protein phosphatase 1 regulatory subunit 15A [Homo sapiens]	1.38
PPP1R32	protein phosphatase 1 regulatory subunit 32 isoform 2 [Homo sapiens]	2.93
PPP1R9A	neurabin-1 isoform 1 [Homo sapiens]	−1.31
PPP2R2B	serine/threonine-protein phosphatase 2A 55 kDa regulatory subunit B beta isoform isoform g [Homo sapiens]	−2.14
PRDM7	probable histone-lysine N-methyltransferase PRDM7 [Homo sapiens]	−2.74
PRKCB	protein kinase C beta type isoform 2 [Homo sapiens]	4.58
PRKD1	serine/threonine-protein kinase D1 isoform 1 [Homo sapiens]	−1.66
PRKN	E3 ubiquitin-protein ligase parkin isoform 1 [Homo sapiens]	−2.37
PROM1	prominin-1 isoform 2 precursor [Homo sapiens]	−3.62
PRRX2	paired mesoderm homeobox protein 2 [Homo sapiens]	−2.58
PSME2	proteasome activator complex subunit 2 [Homo sapiens]	−1.61
PSTPIP2	proline-serine-threonine phosphatase-interacting protein 2 [Homo sapiens]	3.08
PTGES	prostaglandin E synthase [Homo sapiens]	−5.19
PTK7	inactive tyrosine-protein kinase 7 isoform e [Homo sapiens]	−2.20
PTPRT	receptor-type tyrosine-protein phosphatase T isoform 2 precursor [Homo sapiens]	2.76
PTPRZ1	receptor-type tyrosine-protein phosphatase zeta isoform 2 precursor [Homo sapiens]	−2.21
PXYLP1	2-phosphoxylose phosphatase 1 isoform 1 [Homo sapiens]	−1.35
QDPR	dihydropteridine reductase isoform 1 [Homo sapiens]	−1.92
R3HDM2	R3H domain-containing protein 2 isoform 1 [Homo sapiens]	−1.23
RAB17	ras-related protein Rab-17 [Homo sapiens]	−1.40
RAB6D	ras-related protein Rab-6D [Homo sapiens]	4.84
RABGAP1L	rab GTPase-activating protein 1-like isoform B [Homo sapiens]	−1.24
RAD51B	DNA repair protein RAD51 homolog 2 isoform 4 [Homo sapiens]	−1.39
RBPJL	recombining binding protein suppressor of hairless-like protein isoform 2 [Homo sapiens]	−2.44
RETREG1	reticulophagy regulator 1 isoform 1 [Homo sapiens]	−2.25
RFX3	transcription factor RFX3 isoform b [Homo sapiens]	−1.28
RHOJ	rho-related GTP-binding protein RhoJ precursor [Homo sapiens]	−1.34
RIMS1	regulating synaptic membrane exocytosis protein 1 isoform 2 [Homo sapiens]	4.11
RIMS2	regulating synaptic membrane exocytosis protein 2 isoform a [Homo sapiens]	−2.24
RLBP1	retinaldehyde-binding protein 1 [Homo sapiens]	−2.55
RNF125	E3 ubiquitin-protein ligase RNF125 [Homo sapiens]	−2.17
RNF144A	E3 ubiquitin-protein ligase RNF144A isoform 1 [Homo sapiens]	−2.02

RNF182	E3 ubiquitin-protein ligase RNF182 [Homo sapiens]	−2.24
RNF187	E3 ubiquitin-protein ligase RNF187 [Homo sapiens]	−5.55
RNF207	RING finger protein 207 [Homo sapiens]	−4.09
RNF25	E3 ubiquitin-protein ligase RNF25 [Homo sapiens]	1.46
ROBO2	roundabout homolog 2 isoform ROBO2a [Homo sapiens]	−2.38
RPL41	60S ribosomal protein L41 [Homo sapiens]	−1.88
RPS26	40S ribosomal protein S26 [Homo sapiens]	1.55
RPSAP58	ribosomal protein SA pseudogene 58 [Homo sapiens]	−1.64
RUNX2	runt-related transcription factor 2 isoform b [Homo sapiens]	−1.51
RUNX3	runt-related transcription factor 3 isoform 1 [Homo sapiens]	−1.38
S100A4	protein S100-A4 [Homo sapiens]	−2.14
SAMD5	sterile alpha motif domain-containing protein 5 [Homo sapiens]	−1.67
SAT1	diamine acetyltransferase 1 [Homo sapiens]	1.32
SCD	acyl-CoA desaturase [Homo sapiens]	−1.65
SCN9A	sodium channel protein type 9 subunit alpha isoform 2 [Homo sapiens]	−2.53
SDC3	syndecan-3 precursor [Homo sapiens]	−1.63
SEC24D	protein transport protein Sec24D isoform 2 [Homo sapiens]	1.85
SEMA3E	semaphorin-3E isoform 2 [Homo sapiens]	3.72
SEMA6A	semaphorin-6A isoform 1 precursor [Homo sapiens]	−1.96
SEPTIN6	septin-6 isoform B [Homo sapiens]	−1.74
SERPINA3	alpha-1-antichymotrypsin precursor [Homo sapiens]	−1.85
SERPINA5	plasma serine protease inhibitor preproprotein [Homo sapiens]	−2.30
SERPINB2	plasminogen activator inhibitor 2 [Homo sapiens]	4.13
SERPINE1	plasminogen activator inhibitor 1 precursor [Homo sapiens]	1.43
SERTAD1	SERTA domain-containing protein 1 [Homo sapiens]	1.98
SEZ6L	seizure 6-like protein isoform 2 precursor [Homo sapiens]	5.26
SFRP1	secreted frizzled-related protein 1 precursor [Homo sapiens]	−1.79
SFXN3	sideroflexin-3 [Homo sapiens]	−1.68
SGCA	alpha-sarcoglycan isoform 1 precursor [Homo sapiens]	−3.54
SGCD	delta-sarcoglycan isoform 1 [Homo sapiens]	−2.80
SGCZ	zeta-sarcoglycan isoform 2 [Homo sapiens]	2.51
SHOC1	protein shortage in chiasmata 1 ortholog isoform 2 [Homo sapiens]	2.18
SHROOM4	protein Shroom4 [Homo sapiens]	−2.01
SIPA1L1	signal-induced proliferation-associated 1-like protein 1 isoform 2 [Homo sapiens]	−1.58
SLC19A1	folate transporter 1 isoform 2 [Homo sapiens]	−1.89
SLC1A4	neutral amino acid transporter A isoform 2 precursor [Homo sapiens]	−1.44
SLC22A18	solute carrier family 22 member 18 isoform a [Homo sapiens]	−2.73
SLC22A23	solute carrier family 22 member 23 isoform b [Homo sapiens]	−2.05
SLC24A1	sodium/potassium/calcium exchanger 1 isoform 2 [Homo sapiens]	−1.47
SLC24A3	sodium/potassium/calcium exchanger 3 precursor [Homo sapiens]	4.11
SLC24A5	sodium/potassium/calcium exchanger 5 precursor [Homo sapiens]	−2.02
SLC25A24	calcium-binding mitochondrial carrier protein SCaMC-1 isoform 1 [Homo sapiens]	−1.62
SLC26A2	sulfate transporter [Homo sapiens]	−2.30
SLC28A3	solute carrier family 28 member 3 [Homo sapiens]	−2.79
SLC30A2	zinc transporter 2 isoform 1 [Homo sapiens]	−3.52
SLC35F1	solute carrier family 35 member F1 [Homo sapiens]	−1.39
SLC35F4	solute carrier family 35 member F4 isoform b [Homo sapiens]	3.15
SLC39A10	zinc transporter ZIP10 precursor [Homo sapiens]	−1.46
SLC46A2	thymic stromal cotransporter homolog [Homo sapiens]	−1.90
SLC5A4	solute carrier family 5 member 4 [Homo sapiens]	−2.02
SLC5A9	sodium/glucose cotransporter 4 isoform 2 [Homo sapiens]	−3.28
SLC6A15	sodium-dependent neutral amino acid transporter B(0)AT2 isoform 3 [Homo sapiens]	−1.59
SLCO3A1	solute carrier organic anion transporter family member 3A1 isoform 2 [Homo sapiens]	−2.18
SLCO4A1	solute carrier organic anion transporter family member 4A1 [Homo sapiens]	−1.71
SLITRK6	SLIT and NTRK-like protein 6 precursor [Homo sapiens]	−3.06
SMOC2	SPARC-related modular calcium-binding protein 2 isoform 2 precursor [Homo sapiens]	4.70
SMYD2	N-lysine methyltransferase SMYD2 [Homo sapiens]	−1.55
SNIP1	smad nuclear-interacting protein 1 [Homo sapiens]	1.34
SNTB1	beta-1-syntrophin [Homo sapiens]	−2.16
SNX10	sorting nexin-10 isoform 1 [Homo sapiens]	−1.25
SOBP	sine oculis-binding protein homolog [Homo sapiens]	−1.71
SOD1	superoxide dismutase [Cu-Zn] [Homo sapiens]	1.28
SOSTDC1	sclerostin domain-containing protein 1 precursor [Homo sapiens]	−3.55

SOX13	transcription factor SOX-13 [Homo sapiens]	−1.47
SOX5	transcription factor SOX-5 isoform d [Homo sapiens]	−1.56
SOX6	transcription factor SOX-6 isoform b [Homo sapiens]	−1.44
SPATA31C2	putative spermatogenesis-associated protein 31C2 isoform 2 [Homo sapiens]	−2.47
SPATA6	spermatogenesis-associated protein 6 isoform 2 [Homo sapiens]	−1.40
SPRY1	protein sprouty homolog 1 [Homo sapiens]	−1.49
SREBF1	sterol regulatory element-binding protein 1 isoform a [Homo sapiens]	−1.54
SRGAP3	SLIT-ROBO Rho GTPase-activating protein 3 isoform b [Homo sapiens]	1.39
SRXN1	sulfiredoxin-1 [Homo sapiens]	1.62
SSX2B	protein SSX2 isoform b [Homo sapiens]	−2.25
ST3GAL1	CMP-N-acetylneuraminate-beta-galactosamide-alpha-2,3-sialyltransferase 1 [Homo sapiens]	−1.20
ST3GAL4	CMP-N-acetylneuraminate-beta-galactosamide-alpha-2,3-sialyltransferase 4 isoform 2 [Homo sapiens]	−1.28
ST6GAL1	beta-galactoside alpha-2,6-sialyltransferase 1 isoform a [Homo sapiens]	−1.37
ST8SIA1	alpha-N-acetylneuraminide alpha-2,8-sialyltransferase isoform 2 [Homo sapiens]	−2.38
STC1	stanniocalcin-1 precursor [Homo sapiens]	−4.29
STK32A	serine/threonine-protein kinase 32A isoform 1 [Homo sapiens]	−1.36
STOX2	storkhead-box protein 2 [Homo sapiens]	−1.38
SYCP2L	synaptonemal complex protein 2-like [Homo sapiens]	3.26
SYNC	syncoilin isoform 2 [Homo sapiens]	2.08
SYT1	synaptotagmin-1 isoform 1 [Homo sapiens]	−1.28
SYT9	synaptotagmin-9 [Homo sapiens]	−1.53
TARBP1	probable methyltransferase TARBP1 [Homo sapiens]	−1.65
TBC1D5	TBC1 domain family member 5 isoform a [Homo sapiens]	−1.30
TBC1D9B	TBC1 domain family member 9B isoform b [Homo sapiens]	−2.64
TCF7L1	transcription factor 7-like 1 [Homo sapiens]	−2.36
TEF	thyrotroph embryonic factor isoform 2 [Homo sapiens]	−2.07
TENM1	teneurin-1 isoform 1 precursor [Homo sapiens]	−2.41
TENM4	teneurin-4 [Homo sapiens]	−2.04
TET1	methylcytosine dioxygenase TET1 [Homo sapiens]	−2.62
TGFA	protransforming growth factor alpha isoform 2 preproprotein [Homo sapiens]	−2.89
TGFBR3	transforming growth factor beta receptor type 3 isoform b precursor [Homo sapiens]	−2.15
THADA	thyroid adenoma-associated protein isoform a [Homo sapiens]	−1.27
THBS2	thrombospondin-2 precursor [Homo sapiens]	−2.27
THSD4	thrombospondin type-1 domain-containing protein 4 isoform 2 precursor [Homo sapiens]	−1.96
TIPARP	protein mono-ADP-ribosyltransferase TIPARP [Homo sapiens]	1.72
TMEM164	transmembrane protein 164 isoform b [Homo sapiens]	−1.24
TMEM170B	transmembrane protein 170B [Homo sapiens]	−1.39
TMEM18	transmembrane protein 18 isoform 2 [Homo sapiens]	−2.20
TMEM208	transmembrane protein 208 isoform 2 [Homo sapiens]	1.57
TMEM225B	transmembrane protein 225B [Homo sapiens]	2.34
TMEM232	transmembrane protein 232 [Homo sapiens]	−1.92
TMEM255A	transmembrane protein 255A isoform 2 [Homo sapiens]	−2.04
TMEM39A	transmembrane protein 39A [Homo sapiens]	1.30
TMEM40	transmembrane protein 40 isoform a [Homo sapiens]	3.25
TMOD2	tropomodulin-2 isoform b [Homo sapiens]	−2.54
TMTC2	protein O-mannosyl-transferase TMTC2 isoform 2 [Homo sapiens]	−1.62
TMX4	thioredoxin-related transmembrane protein 4 precursor [Homo sapiens]	−1.25
TNC	tenascin precursor [Homo sapiens]	−1.68
TNFAIP3	tumor necrosis factor alpha-induced protein 3 [Homo sapiens]	−1.51
TNFRSF12A	tumor necrosis factor receptor superfamily member 12A precursor [Homo sapiens]	1.79
TNFRSF19	tumor necrosis factor receptor superfamily member 19 isoform 2 precursor [Homo sapiens]	−2.72
TNN	tenascin-N precursor [Homo sapiens]	−1.40
TNS1	tensin-1 isoform 2 [Homo sapiens]	−4.54
TRABD2B	metalloprotease TIKI2 precursor [Homo sapiens]	−2.47
TRAPPC13	trafficking protein particle complex subunit 13 isoform 1 [Homo sapiens]	1.47
TRIM16	tripartite motif-containing protein 16 isoform a [Homo sapiens]	−1.35
TRIM2	tripartite motif-containing protein 2 isoform 2 [Homo sapiens]	−2.93
TRIM62	E3 ubiquitin-protein ligase TRIM62 isoform 2 [Homo sapiens]	−1.49
TRIM66	tripartite motif-containing protein 66 [Homo sapiens]	−1.37
TRIM9	E3 ubiquitin-protein ligase TRIM9 isoform 1 [Homo sapiens]	−1.67
TRPV4	transient receptor potential cation channel subfamily V member 4 isoform e [Homo sapiens]	−2.42
TSHR	thyrotropin receptor isoform 1 precursor [Homo sapiens]	−2.15

TSNAXIP1	translin-associated factor X-interacting protein 1 isoform a [Homo sapiens]	−2.31
TSPYL2	testis-specific Y-encoded-like protein 2 [Homo sapiens]	1.96
TUFT1	tuftelin isoform 2 [Homo sapiens]	1.80
TXNDC8	thioredoxin domain-containing protein 8 isoform a [Homo sapiens]	4.10
TXNIP	thioredoxin-interacting protein isoform 2 [Homo sapiens]	−4.45
TXNRD1	thioredoxin reductase 1, cytoplasmic isoform 5 [Homo sapiens]	1.95
TYMP	thymidine phosphorylase isoform 1 precursor [Homo sapiens]	−2.22
TYR	tyrosinase precursor [Homo sapiens]	−2.17
UBAP1L	ubiquitin-associated protein 1-like [Homo sapiens]	−1.96
UBOX5	RING finger protein 37 isoform c [Homo sapiens]	1.62
UCN2	urocortin-2 preproprotein [Homo sapiens]	−2.03
USP13	ubiquitin carboxyl-terminal hydrolase 13 [Homo sapiens]	−1.25
VAMP7	vesicle-associated membrane protein 7 isoform 2 [Homo sapiens]	2.16
VCAN	versican core protein isoform 2 precursor [Homo sapiens]	−1.32
VGLL3	transcription cofactor vestigial-like protein 3 isoform 2 [Homo sapiens]	1.42
VILL	villin-like protein isoform 2 [Homo sapiens]	−2.17
VLDLR	very low-density lipoprotein receptor isoform b precursor [Homo sapiens]	−1.84
VPS37B	vacuolar protein sorting-associated protein 37B [Homo sapiens]	1.75
VSIG8	V-set and immunoglobulin domain-containing protein 8 precursor [Homo sapiens]	−4.50
WDR25	WD repeat-containing protein 25 isoform a [Homo sapiens]	−1.26
WDR27	WD repeat-containing protein 27 isoform 2 [Homo sapiens]	−1.51
WDR62	WD repeat-containing protein 62 isoform 1 [Homo sapiens]	1.46
WWOX	WW domain-containing oxidoreductase isoform 4 [Homo sapiens]	−2.10
XXYLT1	xyloside xylosyltransferase 1 isoform 2 [Homo sapiens]	−1.64
XYLT1	xylosyltransferase 1 precursor [Homo sapiens]	−3.33
YOD1	ubiquitin thioesterase OTU1 isoform 2 [Homo sapiens]	1.31
YTHDC1	YTH domain-containing protein 1 isoform 1 [Homo sapiens]	−3.11
YWHAE	14-3-3 protein epsilon [Homo sapiens]	−3.85
ZBTB7C	zinc finger and BTB domain-containing protein 7C [Homo sapiens]	−2.32
ZC3H4	zinc finger CCCH domain-containing protein 4 [Homo sapiens]	−1.60
ZCCHC24	zinc finger CCHC domain-containing protein 24 [Homo sapiens]	−1.75
ZFAND2A	AN1-type zinc finger protein 2A isoform 2 [Homo sapiens]	1.63
ZFP82	zinc finger protein 82 homolog isoform 2 [Homo sapiens]	4.84
ZMIZ1	zinc finger MIZ domain-containing protein 1 [Homo sapiens]	−1.21
ZNF114	zinc finger protein 114 isoform 1 [Homo sapiens]	−2.57
ZNF143	zinc finger protein 143 isoform 2 [Homo sapiens]	1.62
ZNF239	zinc finger protein 239 isoform a [Homo sapiens]	−1.96
ZNF334	zinc finger protein 334 isoform c [Homo sapiens]	−1.38
ZNF33A	zinc finger protein 33A isoform c [Homo sapiens]	−1.67
ZNF33B	zinc finger protein 33B isoform 1 [Homo sapiens]	−1.33
ZNF362	zinc finger protein 362 [Homo sapiens]	−1.65
ZNF480	zinc finger protein 480 isoform 2 [Homo sapiens]	−1.70
ZNF521	zinc finger protein 521 isoform 2 [Homo sapiens]	−1.57
ZNF652	zinc finger protein 652 [Homo sapiens]	−1.54
ZNF689	zinc finger protein 689 [Homo sapiens]	−1.77
ZNF699	zinc finger protein 699 [Homo sapiens]	2.75
ZNF704	zinc finger protein 704 isoform 1 [Homo sapiens]	−1.93
ZNRF1	E3 ubiquitin-protein ligase ZNRF1 [Homo sapiens]	−1.80

Table S2. Primer sequences for Real-time PCR analysis.

Primer	Sequences (5`-3`)
Caspase-3-F	CTCGGTCTGGTACAGATGTCGA
Caspase-3-Rev	CATGGCTCAGAAGCACACAAAC
Caspase-8-F	GGTCACTTGAACCTTGGGAA
Caspase-8-Rev	AGGCCAGATCTTCACTGTCC
Caspase-9-F	GTGGACATTGGTTCTGGAGGAT
Caspase-9-Rev	CGCAACTTCTCACAGTCGATG
Bax-F	GCTGGACATTGGACTTCCTC
Bax-Rev	TCAGCCCATCTTCTTCCAGA
Bcl-2-F	CATGTGTGTGGAGAGCGTCAA
Bcl-2-Rev	GCCGGTTCAGGTACTCAGTCA
GAPDH-F	GAAGGTGAAGGTCGGAGTCA
GAPDH-Rev	TTGAGGTCAATGAAGGGGTC
